# DNAzyme Cleavage of CAG Repeat RNA in Polyglutamine Diseases

**DOI:** 10.1007/s13311-021-01075-w

**Published:** 2021-06-23

**Authors:** Nan Zhang, Brittani Bewick, Jason Schultz, Anjana Tiwari, Robert Krencik, Aijun Zhang, Kaho Adachi, Guangbin Xia, Kyuson Yun, Partha Sarkar, Tetsuo Ashizawa

**Affiliations:** 1 Department of Neurology, Neuroscience Program, Houston Methodist Research Institute, Houston, TX USA; 2 Center for Neuroregeneration, Department of Neurosurgery, Houston Methodist Research Institute, Houston, TX USA; 3 Center for Bioenergetics, Department of Neurosurgery, Houston Methodist Research Institute, Houston, TX USA; 4 Department of Molecular and Cell Biology, UC-Berkeley, Berkeley, CA USA; 5 Indiana University School of Medicine-Fort Wayne, Fort Wayne, IN USA; 6 Department of Neurology and Department of Neuroscience, Cell Biology and Anatomy, UTMB Health, Galveston, TX USA

**Keywords:** DNAzyme, Polyglutamine, CAG repeats, Huntington’s disease, Spinocerebellar ataxia, Microsatellite expansion

## Abstract

CAG repeat expansion is the genetic cause of nine incurable polyglutamine (polyQ) diseases with neurodegenerative features. Silencing repeat RNA holds great therapeutic value. Here, we developed a repeat-based RNA-cleaving DNAzyme that catalyzes the destruction of expanded CAG repeat RNA of six polyQ diseases with high potency. DNAzyme preferentially cleaved the expanded allele in spinocerebellar ataxia type 1 (SCA1) cells. While cleavage was non-allele-specific for spinocerebellar ataxia type 3 (SCA3) cells, treatment of DNAzyme leads to improved cell viability without affecting mitochondrial metabolism or p62-dependent aggresome formation. DNAzyme appears to be stable in mouse brain for at least 1 month, and an intermediate dosage of DNAzyme in a SCA3 mouse model leads to a significant reduction of high molecular weight ATXN3 proteins. Our data suggest that DNAzyme is an effective RNA silencing molecule for potential treatment of multiple polyQ diseases.

**Supplementary Information:**

The online version contains supplementary material available at 10.1007/s13311-021-01075-w.

## Introduction

DNAzymes (Dzs) are single-stranded RNA-cleaving DNAs that typically consist of a catalytic core and two flanking RNA-binding arms for target recognition. The catalytic core cleaves a phosphodiester bond in the presence of divalent cations, generating a 5′-product with a 2′,3′-cyclic phosphate at the 3′-end and a 3′-product with a hydroxyl group at the 5′-end. The 8–17 DNAzyme is among the first identified DNAzymes that efficiently cleave RNA substrates [1]. The 15-nucleotide (nt) core has been independently isolated under different cation selections such as Mg^2+^, Mn^2+^, Ca^2+^, Zn^2+^, and Pb^2+^ and shown to twist into a pseudoknot with the two RNA binding arms 70° apart in the crystal structure [2]. Despite their high reprogrammability and versatility for chemical modification and nanoconjugation, the theranostic value and means of delivery are largely unexplored.

Polyglutamine (polyQ) diseases are a group of incurable neurodegenerative disorders caused by CAG repeat expansion in coding regions, including Huntington’s disease (HD), spinocerebellar ataxias (SCA types 1, 2, 3, 6, 7, and 17), dentatorubropallidoluysian atrophy (DRPLA), and spinobulbar muscular atrophy (SBMA) [3]. A toxic gain of function of the expanded polyQ proteins has been proposed to cause a variety of potential pathogenic cellular events, including single- or double-stranded (ss/ds) DNA breaks [4–9], impaired protein homeostasis [10–13], transcription dysregulation [14, 15], protein sequestration [16, 17], disruption of nucleocytoplasm transport and endoplasmic reticulum architecture [16, 18–20], and mitochondrial dysfunction [21–23] in different diseases. In addition, the expanded RNA can also give rise to noncanonical repeat-associated non-ATG (RAN) translation products [24–26]. Thus, ridding cells of the mutant polyQ RNA/protein has become a major disease intervention and its success is thought to ameliorate any downstream pathomechanistic defects.

Significant progresses have been made through CRISPR-Cas genome editing and antisense oligonucleotide (ASO) therapies in many polyQ diseases, especially in HD, SCA2, and SCA3 [27–38]. Efforts towards engineering high-fidelity Cas proteins with various controller modules, split Cas proteins for efficient viral packaging, and non-viral delivery approaches are underway to improve precision and safety [39–42]. ASOs have been successfully tested in HD, SCA1, SCA2, SCA3, and SCA7 cells or mouse models and with HD ASO entering the Phase III clinical trial [30, 33, 34, 36, 37, 43–45]. The efficacy of ASO may vary from case to case and may largely depend on RNase H activity, accessibility of target sites, design chemistry, and bioavailability in target tissue. Observations from clinical trials and animal studies suggest that targeting both mutant and normal alleles of the polyQ protein does not associate with any pathology or deleterious side effects. In addition, no off-target effects were detected using CAG complementary ASOs in HD and SCA3 patient cells or HD Q175 mice [46–48]. The above observations have clearly outlined the possibility and benefit of using a single repeat-based agent to reduce mutant polyQ RNA and protein load across multiple diseases.

Since the 8–17 core cleaves the phosphodiester bond between ribonucleotides A and G, we designed an 8–17 DNAzyme that binds to and cleaves CAG repeat RNA. We demonstrate that the designer DNAzyme cleaves its target in the presence of different ions and under physiological concentrations. The chemically stabilized DNAzyme retains the catalytic activity and knocks down or eliminates mutant polyQ RNA or protein load in cells from several polyQ disease origins and high molecular weight (HMW) ATXN3 proteins in a SCA3 mouse model.

## Materials and Methods

### General Methods and DNA

Unmodified DNA oligonucleotides were purchased from Sigma-Aldrich. PNA oligonucleotides were purchased from PNA Bio. LNA oligonucleotides were purchased from Qiagen. Q5 high-fidelity DNA polymerase (New England Biolabs) was used for routine PCR amplification. Restriction digestion and ligation were performed with FastDigest enzymes and T4 DNA Ligase Kit (Thermo Fisher Scientific), respectively. DNA plasmids were propagated in chemically competent *Escherichia coli* Stbl3 (Thermo Fisher Scientific) and sequenced at GENEWIZ.

HEK293 and SH-SY5Y cells were cultured at 37 °C with 5% CO_2_ in Dulbecco’s modified Eagle’s medium (DMEM, GIBCO) containing 10% non-inactivated fetal calf serum (FBS, GIBCO) and 100 U/ml penicillin–streptomycin (P/S, GIBCO). Normal (GM23973) and patient-derived SCA1 (GM06927, 52/29 CAG repeats) and SCA3 (GM06151, 74/24 CAG repeats) fibroblast cells were purchased from Coriell Cell Repositories and cultured in DMEM supplemented with 10% FBS and 1% P/S. Fibroblasts used in this study are no more than 2 passages different across cell lines and are all below 15 passages for nucleofection. BMECs (brain microvascular endothelial cells, gift of Dr. Kyuson Yun) were cultured on plates coated with 2 µg/cm^2^ fibronectin at 37 °C in Medium 199 (Gibco) containing 5% NuSerum, 5% FBS, and 1% P/S. Normal WTC11 iPSCs (GM25256) were purchased from Coriell Cell Repositories. Different iPSC lines were cultured in TeSR™-E8™ (STEMCELL Technologies) on plates or round coverslips coated with Matrigel (Corning).

The expanded *ATXN1* (gift of Dr. Huda Zoghbi), *TBP* (gift of Dr. Shihua Li), *ATXN7* (gift of Dr. Harry Orr), and *AR* (Addgene #28,235) genes were cloned from their parental backbones into pCMV-(DYKDDDDK)-N vector to acquire a FLAG tag (Clontech). The pEGFP-Q74 (for HTTex1, #40,262), pEGFP-C1-ATXN3-Q28 (#22,122), and pEGFP-C1-ATXN3-Q84 (#22,123) plasmids were purchased from Addgene.

### Animals

All animal procedures were approved by the Houston Methodist Institutional Animal Care and Use Committee and conducted in compliance with the National Institutes of Health guidelines for the use of experimental animals. Genotyping was performed using tail biopsy DNA isolated prior to weaning. PCR was performed using Qiagen DNeasy Blood and Tissue Kit and primers recommended by the Jackson Laboratory to amplify a fragment of the *ATXN3* transgene. Animals were matched for age, sex, and weight for this study. Six-month old animals were injected with either saline or DNAzyme and brain tissues were harvested after 1 month for imaging and biochemical analyses. Before euthanization, blood was drawn and analyzed for clinical chemistry and hematology at the MD Anderson core facility. Different experimental stages—such as injection, brain harvest, and biochemical assays—were carried out by different personnel to ensure data reproducibility.

### In vivo Imaging System (I.V.I.S.) and Stereotaxic Injection

I.V.I.S. imaging was performed using a Caliper Xenogen 200 system, and images were normalized using the Living Image Software.

“Naked” DNAzyme resuspended in PBS was stereotaxically administered into the right lateral ventricle. Intracerebroventricular (i.c.v.) injections were performed on mice under intraperitoneal ketamine anesthesia. For each i.c.v. injection, a small incision was made to expose the Bregma and a small hole was drilled at the coordinates pre-determined by the NeuroStar software (mediolateral + 1 mm; anterior–posterior − 0.3 mm; dorsoventral − 2.5 mm). Four hundred μg LNA8-17Dz9 was delivered at an infusion rate of 4 turns/min using a Hamilton syringe on a Global Biotech stereotaxic instrument. After the injection was complete, the needle was slowly retracted at a rate of 1 mm/min and animals were sutured and supplied with a painkiller.

### RNA In vitro Transcription and Cleavage Assay

CAG_x30_ and CAGx42 were cloned into pcDNA3.1-hygro + at NheI and HindIII sites, linearized at the EcoRV site, and in vitro transcribed at 30 °C overnight using HiScribe T7 High Yield RNA Synthesis Kit (NEB) with approximately 500 ng of DNA input. The transcribed RNA was digested with TURBO DNase I at 37 °C for 30 min before column purified using RNA Clean and Concentrator (Zymo Research).

RNA cleavage assays were performed in 1 × Dz digestion buffer (50 mM Tris–HCl pH 7.5, 150 mM NaCl and varying concentrations of Mg^2+^ or Ca^2+^) with 100 nM target RNA and 500 nM 8-17Dz at 37 °C for the indicated time. Samples were quenched in 2 × TBE-Urea Loading Buffer (Bio-Rad) mixed with 3 × formamide dye (100% formamide, 0.5 M EDTA), boiled at 95˚C for 5 min, and loaded on 10% TBE-Urea gels. After running at 200 V for approximately 1 h, RNA cleavage was detected in SYBR Gold (Invitrogen, 1:50,000) on an Azure c400 Biosystem.

### Transfection

HEK293 or SH-SY5Y cells were seeded at 700,000 per well (6-well format), approximately 16 h before transfection. Transfection was performed with 2 µg plasmid DNA ± 200 pmol 8-17Dz in the presence of 15 µl Lipofectamine 2000 (Invitrogen) according to the manufacturer’s instruction.

Fibroblasts were nucleofected using the P2 Primary Cell 4D-Nucleofector X Kit (100 µl format) on a 4D-Nucleofactor X unit (Lonza) according to the manufacturer’s instruction. One million cells combined ± 200 or 300 pmol DNAzyme were nucleofected under the program “CZ167” and plated in pre-warmed medium in each well. Analyses (immunoblot/IF/functional assays) were performed 48 h post transfection.

Each 100-pmol DNAzyme was transfected with 1.5 µl Lipofectamine Stem Reagent (Thermo Fisher Scientific) into iPSCs or differentiating iNeurons according to the manufacturer’s protocol.

### RNA Isolation and Quantitative RT-PCR (qRT-PCR)

Total RNA was isolated using RNeasy Mini Kit (Qiagen), digested with TURBO DNase I (Thermo Fisher Scientific), and purified using RNA Clean and Concentrator (Zymo Research) according to the manufacturer’s protocol. One microgram of input RNA was reverse transcribed using iScript cDNA Synthesis Kit (Bio-Rad). PCR amplification was performed with 2.5 µl of diluted cDNA (1:3 dilution) on a CFX96 Real-time System (Bio-Rad). RNA expression was normalized against that of *GAPDH*, and the relative normalized expression was calculated using CFX Manager 3.1 (Bio-Rad).

### DCL64 Liposome Packaging

1,2-Dipalmitoyl-sn-glycero-3-phosphocoline (DPPC) and cholesterol were purchased from Avanti Polar Lipids and poloxamer L64 was purchased from Sigma-Aldrich. DCL64 liposomes were prepared by mixing DPPC, cholesterol, and poly(ethylene glycose)-block-poly(propylene glycol)-block-poly(ethylene glycol) L64 (poloxamer L64) at a 5:3:7 weight ratio at room temperature. DNAzyme was mixed with DCL64 at a 1:10 weight ratio at room temperature. All DCL64 liposomes were prepared in Wheaton glass vials and mixed with excess tert-butanol at room temperature prior to freezing at − 80 °C overnight. The DCL64 liposomes were then lyophilized overnight, using a FreeZone 2.5 lyophilizer (Labconco), until a thin film had formed, and there was no evidence of liquid within the glass vial. DCL64 liposomes not needed for use immediately were stored at − 20 °C. The DCL64 liposomes were reconstituted with sterile 1 × PBS to the desired concentration immediately before use.

### DNA Tetrahedron Assembly and Packaging

PAGE-purified DNA oligoneucleotides (Table S3) were purchased from Sigma-Aldrich. DNA tetrahedra were generated by mixing equimolar quantities (1 µM) of each strand (± DNAzyme) in 1 × TM buffer (20 mM Tris–HCl pH 7.5, 50 mM MgCl_2_-H_2_O), and the mixture was slowly cooled from 95 to 25 °C over 2 h. The assembled structure was characterized by 1% agarose gel and 4% native PAGE gel. Two assembly reactions were pooled (equivalent to 200 pmol of DNAzyme) for cell delivery.

### Protein Isolation and Immunoblot

Proteins were isolated from cells 48 h post transfection and lysed in Pierce IP Lysis Buffer (Thermo Fisher Scientific) supplemented with cOmplete Protease Inhibitor Cocktail (Roche) and PhosSTOP (Sigma-Aldrich) at 4 °C for 30 min. Soluble fractions were collected after centrifugation at 13,200 rpm at 4 °C for 15 min and quantified using Pierce BCA Protein Assay Kit (Thermo Fisher Scientific).

For immunoblot analysis, approximately 40 µg of proteins were denatured in 4 × Laemmli Sample Buffer (Bio-Rad) and β-mercaptoethanol at 95 °C for 5 min before loaded on 4–15% SDS-PAGE gels (Bio-Rad). Proteins were transferred onto PVDF membranes (Bio-Rad) using a Mini Trans-Blot Electrophoretic Transfer Cell (Bio-Rad) at 100 V for approximately 1.5–2 h at 4 °C. Membranes were incubated with blocking buffer (5% Blotting-Grade Blocker, Bio-Rad, in 1 × TBST, 0.1% Tween 20) at room temperature for 1 h, followed by primary antibody incubation at 4 °C overnight and secondary antibody incubation at room temperature for 1 h. Chemiluminescent signals were detected using SuperSignal West Pico Chemiluminescent Substrate (Thermo Fisher Scientific) on an Azure c400 Biosystem and quantified using ImageJ.

Right cortical tissues from mouse brain were flash frozen for protein analysis. Total protein lysate was extracted using mechanical homogenization in 500 μl RIPA buffer containing cOmplete mini protease inhibitor cocktail and PhoStop phosphatase inhibitor. Homogenate was incubated at 4 °C with agitation for 2 h prior to clearing the debris via centrifugation at 12,000 rpm for 20 min at 4 °C. Supernatants were used for immunoblotting analysis.

Primary antibodies used in this study include mouse anti-ATXN1 (2F5, 1:1,000, Thermo Fisher Scientific), rabbit anti-ATXN2 (NBP1-90,063, 1:200, Novusbio), mouse anti-ATXN3 (MAB5360, 1:1,000, Millipore), rabbit anti-ATXN3 (13,505–1-AP, 1:500, Proteintech), mouse anti-FLAG (M2, 1:1,000, Sigma-Aldrich), mouse HRP-conjugated GAPDH (HRP-60004, Proteintech), mouse anti-GFP (GF28R, 1:1,000, Thermo Fisher Scientific), mouse anti-polyglutamine (MAB1574/5TF1-1C2, 1:1,000, Millipore), rabbit anti-Oct4 (#2750, 1:200, Cell Signaling Technology), rabbit anti-Sox2 (D6D9, 1:200, Cell Signaling Technology), rabbit anti-Nanog (D73G4, 1:200, Cell Signal Technology), mouse anti-8-OHdG (E-8, 1:200, Santa Cruz Biotechnology), rabbit anti-ATXN7 (PA1-749, 1:1,000, Thermo Fisher Scientific), mouse anti-TBP (1TBP18, 1:1,000, Thermo Fisher Scientific), and mouse anti-p62/SQSTM1 (D-3, 1:500, Santa Cruz Biotechnology).

### iNeuron Differentiation and Harvest

iNeurons were differentiated as previously described [49]. Briefly, WTC11 iPSCs with integrated hNGN2 were cultured on 6-well plates coated with Matrigel and induced in cortical neuron culture medium (DMEM/F12 with HEPES, 1 × B27 supplement, doxycycline 2 µg/ml). On day 3, cells were individualized by Accutase (STEMCELL Technologies), seeded on Matrigel coated 6-well plates, and transfected using Lipofectamine Stem Transfection Reagent with HTTex1-Q74 ± DNAzyme (total DNA: LipoStem ratio is 1: 2) according to the manufacturer’s protocol. Cells were harvested for immunoblot or imaging on day 5 and day 7.

### Generation of SCA3 iPSC

The study was approved by the University of Florida Institutional Review Board. The SCA3 subject was provided with the approved informed consent. Skin biopsy was performed by punch biopsy (6 mm in diameter) under local anesthesia. The skin specimens were placed in sterile DMEM medium supplemented with 20% FBS and 1% P/S at room temperature for transport to the lab. Biopsy specimens were processed into 0.5-mm cubes and placed into duplicate 25 cm^2^ flasks. The explants were air-dried for 30 min and 12 ml of primary culture medium (DMEM with 20% FBS) was added to the flask. The flasks were placed in a 37 °C 5% CO_2_ incubator. The medium was replenished after 7 days. When fibroblasts from adjacent explants started to merge, the flasks were treated with 0.05% Trypsin/EDTA and passed to a 75 cm^2^ flask. These cells were designated as passage 1. Passage 3 fibroblasts were used for reprogramming.

Reprogramming was performed by a non-integrating method using CytoTune Sendai Reprogramming Kit (Thermo Fisher Scientific) on the fibroblasts according to the manufacturer’s protocol as previously published [50]. Isolated iPSC clones were cultured on either vitronectin- or Matrigel-coated plates in TeSR-E8 medium (STEMCELL Technologies).

### Fluorescent Microscopy and Immunofluorescence (IF)

To evaluate DNAzyme’s cellular uptake, BMEC or SH-SY5Y cells were incubated with naked or DCL64-packaged Cy3-LNA8-17Dz9 for 48 h, washed five times with PBS (5 min each), fixed in 4% paraformaldehyde (PFA) solution in PBS (Thermo Fisher Scientific) for 10 min, permeabilized in 0.2% Triton X-100 in PBS for 10 min, and mounted in Mounting Medium with DAPI for Fluorescence (Vectorshield). Images were taken on a Nikon A1 Confocal Imaging system.

IF was carried out 48 h post transfection. Fibroblasts, iPSCs, or iNeurons on coverslips were washed with PBS, fixed in 4% PFA, permeabilized in 0.2% Triton X-100 in PBS, and blocked in 5% BSA in PBST (0.05% Tween 20). Primary antibody incubation was carried out at 4 °C overnight, followed by fluorophore-tagged secondary antibody incubation at room temperature for 1 h before mounting in DAPI medium. A Nikon A1 Confocal Imaging system was used.

After brain harvest, sagittal sections of all mouse brains were fixed in 4% PFA for 48 h, cryoprotected in a sucrose gradient, and embedded in OCT. Cryostat sections from OCT embedded tissue were cut at 10 μm thickness and mounted on slides. Sections were fixed in 4% PFA, washed in PBS, and blocked in 5% normal goat serum, 0.2% Triton X-100 in PBS. Sections were then incubated in mouse anti-GFAP antibody (1:500, sc-33673, Santa Cruz Biotechnology) at 4 °C overnight. After washing, the sections were incubated in secondary antibody, washed in PBS, and mounted in Mounting Medium with DAPI (Vectorshield). Slides were imaged with a Nikon A1 Confocal Imaging system.

### Cell Proliferation Assay

Cell proliferation was assessed using the Aqueous One Solution Cell Proliferation Assay (Promega). In brief, 5000 cells (± nucleofected DNAzyme) were plated in each 96 well and treated with 20 µl MTS at 37 °C for 3 h. Absorbance at 490 nm was recorded on a PerkinElmer EnSpire Multimode Plate Reader.

### Mitochondrial Membrane Potential Assay

Mitochondrial membrane potential was measured using the JC-10 dye according to the manufacturer’s protocol (G-Biosciences). In brief, 5000 cells (from the same nucleofection used for cell proliferation assays) were plated in each of the 96 wells, incubated with 100 µl JC-10 solution (1: 200 dilution) for 20 min at 37 °C, washed twice with 1 × MMP solution (1: 5 dilution), and recorded at excitation/emission 535 nm/595 nm and 485 nm/535 nm on the EnSpire plate reader. The red-to-green fluorescence ratio was used for plotting the graph.

### RNAseq

Normal fibroblasts were nucleofected without or with 200 pmol LNA8-17Dz9 (mock vs Dz200) as described above (2 biological replicates for each). After 48 h, total RNA was isolated using RNeasy Mini Kit (Qiagen), treated with DNase I, and purified using RNA Clean and Concentrator (Zymo Research). The quality of RNA was assessed using Agilent 2100 at Novogene; all samples have a RIN number > 8. The library was constructed at Novogene and RNA-seq was carried out with Illumina NovaSeq 6000 PE150 at 20 M depth. Raw reads were processed with fastp (version 0.20.1), mapped to the human reference genome (GRCh38 and Ensembl gene annotation) with STAR (version 2.7.6a), sorted with SAMtools (version 1.9), and finally counted with HTSeq-count (version 0.11.2) under the miniconda environment. Raw counts were normalized to counts per million mapped reads (CPM), and subsequently FDR, p-value and LogFC were calculated with Degust. Genes were considered significantly differentially expressed at FDR < 0.25 and p-value < 0.05, or | Log2FC |> 1.5 and FDR < 0.05.

### Statistical Analysis and Image Processing

Statistical analyses of all data were performed using *t*-test in GraphPad Prism Version 7.03 (*P < 0.05, **P < 0.01, ***P < 0.001, ****P < 0.0001).

Quantification of p62 aggresome/cell was performed in Fiji ImageJ. p62 foci were counted using “Find maxima” with the threshold set to 60. Cells were counted in 8-bit images with threshold set to 30–255 and particle size set to 60–infinity.

## Results

### Designer DNAzyme 8–17 (8-17Dz) Cleaves CAG Repeat RNA Biochemically

**Fig. 1:**
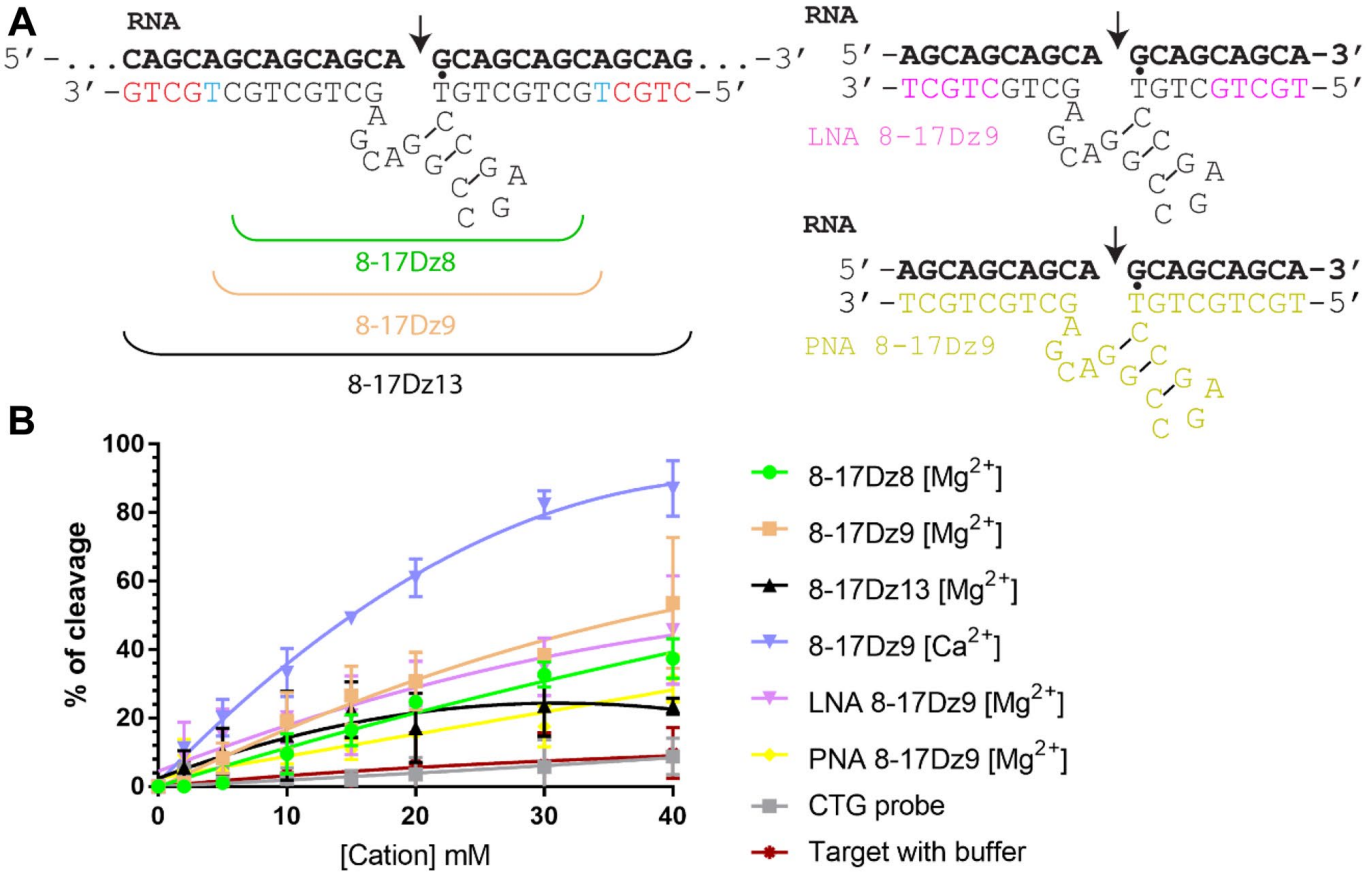
DNAzyme design and biochemical activity. **A** Designs of 8-17Dzs with different RNA binding arms’ lengths. Locked nucleic acids (LNAs) are highlighted in magenta, and peptide nucleic acids (PNAs) are highlighted in yellow. The T·G mismatch is indicated. **B** Biochemical RNA cleavage assays at 37 °C for 1 h under different cation concentrations (*n* = 3). A CTG probe complementary to the CAG RNA repeats were used as a negative control

As a proof-of-concept experiment, three 8-17Dzs with varying lengths of CAG complementing arms were initially designed and tested for cleavage efficiency of in vitro transcribed CAG_x30_ RNA (Fig. 1A). Although the highest activity of the 8-17Dz core is normally observed in the presence of Pb^2+^ [51], we decided to initially screen with Mg^2+^—one of the most abundant metal ions in biological fluids. Among the three 8-17Dz designs, the 8-17Dz with 9-nt-binding arms (8-17Dz9) exhibits the highest RNA cleavage activity across the tested range of Mg^2+^ concentrations over 1 h (Figs. 1B and S1). Additional alterations of RNA-binding arm’s length changed the catalytic property of 8-17Dz to different extents (Fig. S2). Further analysis using Ca^2+^ as a cofactor shows superior RNA cleavage efficiency compared to Mg^2+^ (Figs. 1B and S1). To test if the 8-17Dz9 could work under low Mg^2+^ concentrations, RNA cleavage was assayed in the presence of 5 mM Mg^2+^ (Fig. S3). Direct and indirect measurement of total cellular Mg^2+^ concentration by various techniques consistently indicates that total Mg^2+^ ranges between 17 and 20 mM in mammalian cells [52–54]. Although the cytosolic Mg^2+^ concentration in the occipital lobes of the human brain was estimated close to 200 µM [55], the Mg^2+^ concentration in the rest of the brain is largely uninvestigated. In addition, the majority of Mg^2+^ ions are complexed to a broad spectrum of biomolecules, such as nucleotides, and Mg^2+^ is an important counterion for stabilizing DNA (1 to 2 M counterions for distances between the DNA helix axes of 2–4 nm [56]). It is possible that DNAzyme functions as a Mg^2+^ sink and carries out catalysis under physiologically relevant influx/efflux of Mg^2+^. The intracellular Ca^2+^ ions (approximately 100 nM [57]) and other divalent cations may further contribute to DNAzyme’s activity. As shown in Fig. S3, approximately 50% of the target CAG_x30_ RNA were cleaved by 8-17Dz9 in 5 h at 5 mM Mg^2+^. In agreement with our observation, the same 8–17 catalytic core has been successfully used to detect physiological Ca^2+^/Mg^2+^ levels in undiluted human blood serum [58]. Taken together, we demonstrate that the repeat-based 8-17Dz9 efficiently cleaves CAG RNA biochemically under low ionic strength and is activated by both Ca^2+^ and Mg^2+^ ions, potentially generating a compounding effect on CAG RNA cleavage in cells.

### Locked Nucleic Acid Modification Retains 8-17Dz’s Catalytic Activity

Unmodified DNAzyme is likely degraded by cellular nucleases; increased stability and affinity towards its target RNA may offer persistent therapeutic benefit in cells. Among the repertoire of chemical modifications, we chose the locked nucleic acid (LNA) and peptide nucleic acid (PNA) for base substitutions in 8-17Dz9 due to their strong binding affinity and incompatibility with RNase H [59–62]. We substituted the terminal five nucleotides of each binding arm to LNA to create the LNA8-17Dz9 and all nucleic acids to PNA to create the PNA8-17Dz9 (Fig. 1A). To test if these modifications could affect DNAzyme function, we performed RNA cleavage assays at both high and low Mg^2+^ concentrations. The LNA8-17Dz9 retains comparable catalytic activity to that of the unmodified 8-17Dz9 (Figs. 1B and S3). PNA8-17Dz9 showed reduced RNA cleavage compared to the unmodified 8-17Dz9 (Figs. 1B and S3), possibly due to unfavorable folding of the peptidyl catalytic core, as global folding efficiency of DNAzyme has been linked to catalytic activity [63]. We further compared the DNAzyme’s cleavage efficiency on two different in vitro transcribed CAG repeat lengths, CAGx30 and CAGx42 (Fig. S4). The LNA8-17Dz9 biochemically cleaves both RNA repeats with similar catalytic profiles.

### LNA8-17Dz9 Significantly Knocks Down or Eliminates Multiple Mutant polyQ Proteins in HEK293 Cells

**Fig. 2:**
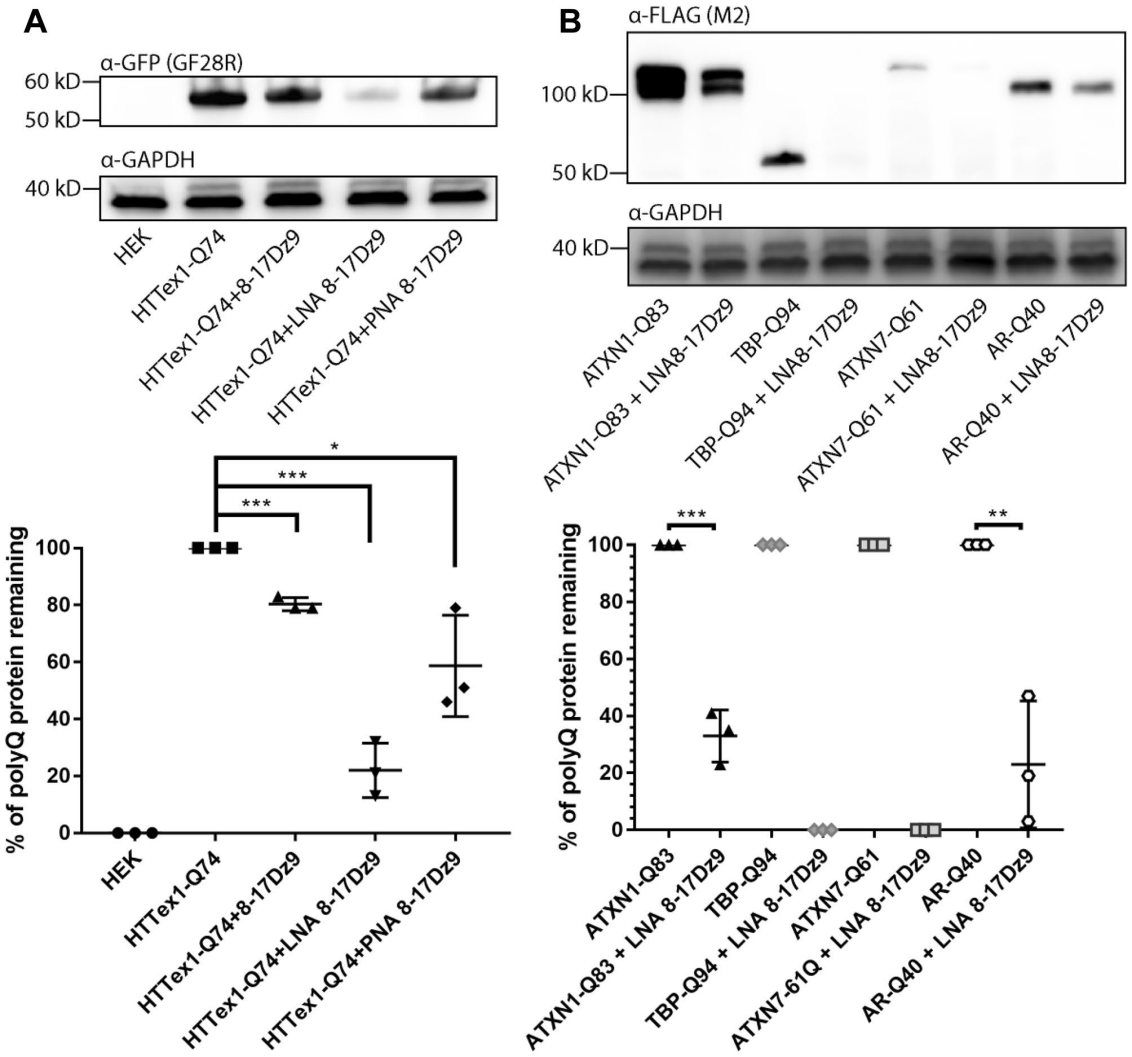
DNAzyme effectively knocks down or eliminates an array of expanded polyQ proteins. **A** HTTex1-Q74 protein knockdown using three 8-17Dz designs (200 pmol each, or 80 nM) in HEK293 cells. **B** Mutant ATXN1-Q83 (both bands were used for quantification), TBP-Q94, ATXN7-Q61, and AR-Q40 were significantly reduced or eliminated by 200 pmol (80 nM) LNA 8-17Dz9 in HEK293 cells

Huntington’s disease is the most common inherited neurodegenerative disease and is caused by CAG repeat expansion in the first exon of Huntingtin (HTTex1 [64],). The mutant HTT (mutHTT) undergoes extensive autolysis and produces exon 1 fragments that aggregate in neuronal nuclei of brain tissue [65]. To test if DNAzyme could knock down mutHTTex1, we co-transfected an HTTex1 fragment containing 74Qs (HTTex1-Q74) with the three 8-17Dz9 designs in HEK293 cells for 48 h. We observed the highest *HTTex1-Q74* RNA knockdown by LNA8-17Dz9, followed by unmodified 8-17Dz9 and PNA8-17Dz9 (Fig. S5). At a protein level, the LNA8-17Dz9 showed the highest knockdown, followed by PNA8-17Dz9 and unmodified 8-17Dz9 (Fig. 2A). We further demonstrate that the observed protein knockdown is not due to competition of DNAzyme with polyQ plasmids during co-transfection by using a control DNA fragment of similar length (Fig. S6). The observed large variability in PNA8-17Dz9 transfected cells could be due to inconsistent packaging between the neutral amide PNA backbone and the cationic transfection lipids. To compare RNA silencing efficiency of our catalytic DNAzyme and RNase H–based antisense oligonucleotide, we designed an ASO containing the same binding arms and LNA modifications without the 8–17 catalytic loop. By targeting eGFP-HTTex1-Q74 in HEK293 cells, we show that LNA8-17Dz9 is approximately 10% more efficient at clearing the expanded polyQ proteins than the ASO counterpart (Fig. S7). Given that LNA8-17Dz9 is most effective in reducing RNA and protein load of mutHTTex1, it was used for the subsequent experiments.

CAG expansion in ataxin 1 (ATXN1), TATA binding protein (TBP), ataxin 7 (ATXN7), and androgen receptor (AR) causes SCA1, SCA17, SCA7, and SBMA, respectively. Each mutant polyQ protein was cloned to acquire a FLAG tag and subsequently co-transfected with LNA8-17Dz9 in HEK293 cells for immunoblot analysis. LNA8-17Dz9 completely eliminates TBP-Q94 and ATXN7-Q61 protein expression and significantly reduces ATXN1-Q83 and AR-Q40 protein levels by approximately 80% (Fig. 2B). Based on our calculated dosage of DNAzyme and cell density, we estimate that between 8 an 12 million DNAzyme molecules are available for intracellular targeting, if a 50% transfection is achieved. To evaluate allele specificity by LNA8-17Dz9, we co-transfected the DNAzyme with either ATXN3-Q28 or ATXN3-Q84 (mutant ATXN3 causes SCA3) in HEK293 cells. LNA8-17Dz9 completely eliminates both proteins (Fig. S8). In the subsequent competitive cleavage experiment, an equal weight of ATXN3-Q28 and ATXN3-Q84 plasmids was co-transfected with LNA8-17Dz9 for immunoblotting. When both proteins are present in HEK293 cells, we did not observe preferential targeting of ATXN3-Q84 over Q28 (Fig. S8). Taken together, the LNA8-17Dz9 efficiently clears a panel of mutant polyQ proteins in HEK293 cells.

### LNA8-17Dz9 Significantly Reduces polyQ Protein Load in Neuroblastoma Cells and iNeurons

**Fig. 3:**
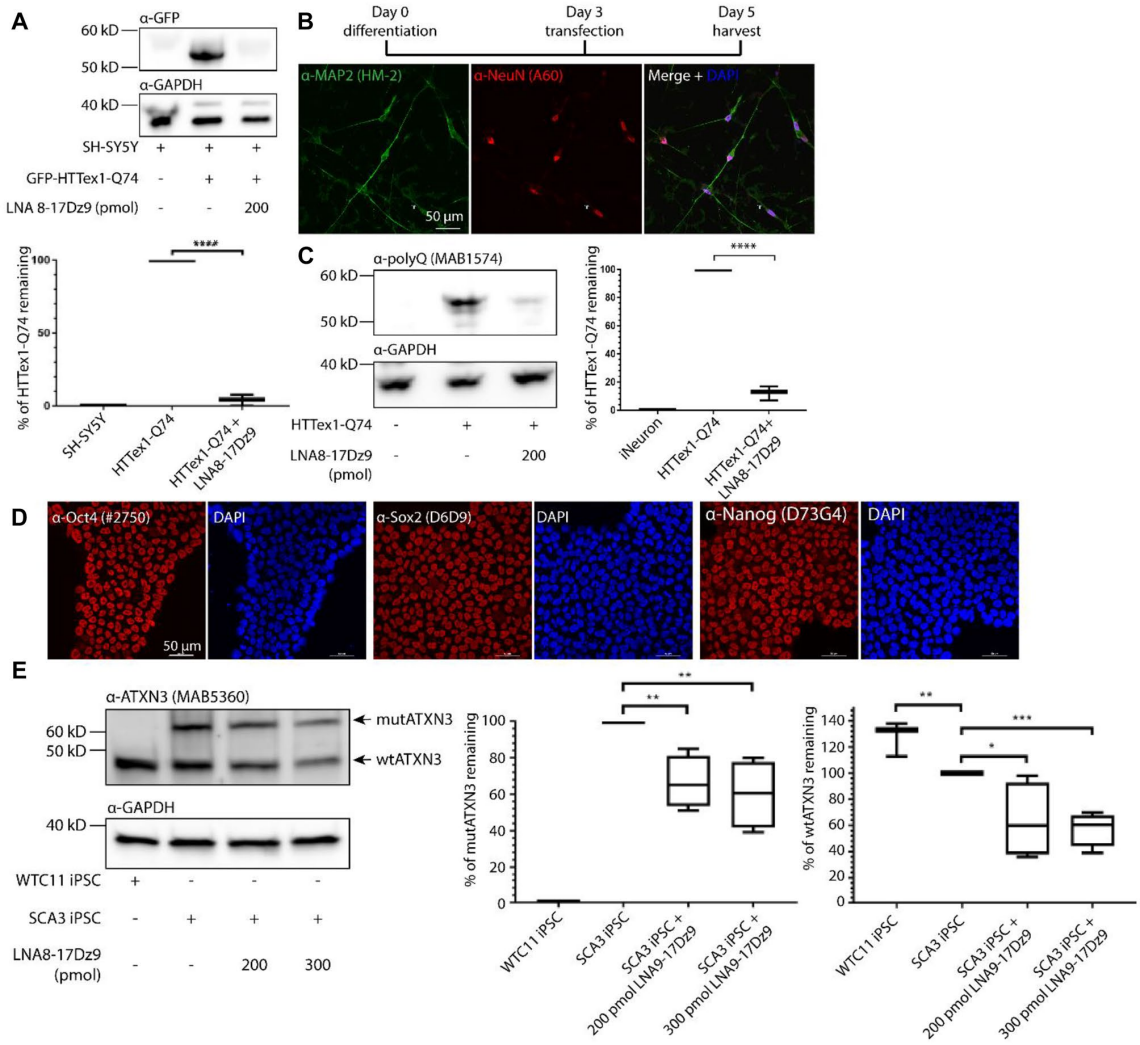
DNAzyme significantly reduces polyQ protein targets in neuronal cells and iPSCs. **A** HTTex1-Q74 protein is significantly reduced in SH-SY5Y cells co-transfected with 200 pmol (80 nM) LNA8-17Dz9. **B** iNeuron differentiation scheme and immunofluorescence (IF) of 5-day differentiated iNeurons against neuronal markers NeuN and MAP2 (with antibody clone numbers indicated in brackets, *n* = 3). **C** Immunoblot and quantification of transfected HTTex1-Q74 levels with or without 200 pmol (80 nM) LNA8-17Dz9 treatment in 5-day differentiated iNeurons (*n* = 3). **D** IF of pluripotency markers in SCA3 iPSCs (*n* = 2). **E** Immunoblot and quantification of mutant and wildtype ATXN3 in SCA3 iPSCs treated with 200 or 300 pmol (80 or 120 nM) LNA8-17Dz9 (*n* = 4). The quantity of mutATXN3 in SCA3 iPSC (without treatment) was used as 100% for normalization

To test if LNA8-17Dz9 could work in neuron-like cells, HTTex1-Q74 and LNA8-17Dz9 were co-transfected into SH-SY5Y neuroblastoma cells. We observed a > 90% target protein knockdown (Fig. 3A). Striatal and cortical neurons exhibit prominent cell loss in postmortem HD brain tissues [66]. To test efficacy in neurons, we utilized a wild-type (wt) human iPSC line stably expressing a doxycycline-inducible human neurogenin 2 (WTC11-hNGN2) at the safe-harbor *AAVS1* locus [67]. Upon induction, the WTC11-hNGN2 iPSCs rapidly differentiate into cortical excitatory neurons (iNeurons) [49, 67, 68]. On a 5-day differentiation scheme, the iNeurons produced dendrites and expressed neuronal markers NeuN and MAP2 (Fig. 3B). Co-transfection of HTTex1-Q74 and 200 pmol LNA8-17Dz9 on day 3 reduced the target protein load by 90% (Fig. 3C). On a 7-day differentiation scheme (transfection on day 3 and harvest on day 7), the iNeurons lost the transgene expression entirely due to the nature of transient transfection (Fig. S9). Taken together, the LNA8-17Dz9 reduces approximately 90% of the polyQ protein load in neuroblastoma cells and iPSC-derived cortical neurons.

### LNA8-17Dz9 Targets Both Normal and Expanded ATXN3 Alleles Without Affecting p62-Aggresome Levels in SCA3 iPSCs

SCA3 is the most common inherited ataxia and is caused by a CAG repeat expansion in the *ATXN3* gene [69]. Although its role as a deubiquitinase has been associated with aggresome formation, autophagy, antiviral response, and transcription-coupled DNA repair [7, 69–74], different *Ataxin3*-knockout (KO) mice exhibit normal viability and fertility, suggesting that its function may not be essential [75, 76]. Thus, lowering the expression of both wild-type and mutant ATXN3 may be well tolerated. We have generated a patient-derived SCA3 iPSC line that expresses pluripotency markers Oct4, Sox2, and Nanog and has a repeat size close to CAG_x80_ (Figs. 3D and S10). Transfection of 200 pmol or 300 pmol of LNA8-17Dz9 showed similar knockdown of both wtATXN3 and mutATXN3 proteins by approximately 40% (Fig. 3E); this non-allele-specific knockdown in iPSCs is consistent with observations in the overexpression system (Fig. S8). The autophagy-linked ubiquitin-binding shuttle protein p62 (SQSTM1) is known to co-localize with both wt and mutATXN3 proteins and promote perinuclear aggresome formation [33, 74, 77–79]. The non-allele-specific knockdown of ATXN3 in iPSCs did not show significant differences in p62-aggresome formation from untreated SCA3 iPSCs or normal WTC11 iPSCs (Fig. S11). Taken together, the LNA8-17Dz9 effectively lowers both wild-type and mutant ATXN3 to a similar extent in SCA3 iPSCs without affecting aggresome formation and by inference p62-dependent autophagy.

### Allele-Specific Targeting of Mutant polyQ Protein Can Be Achieved in Patient-Derived Fibroblasts

**Fig. 4:**
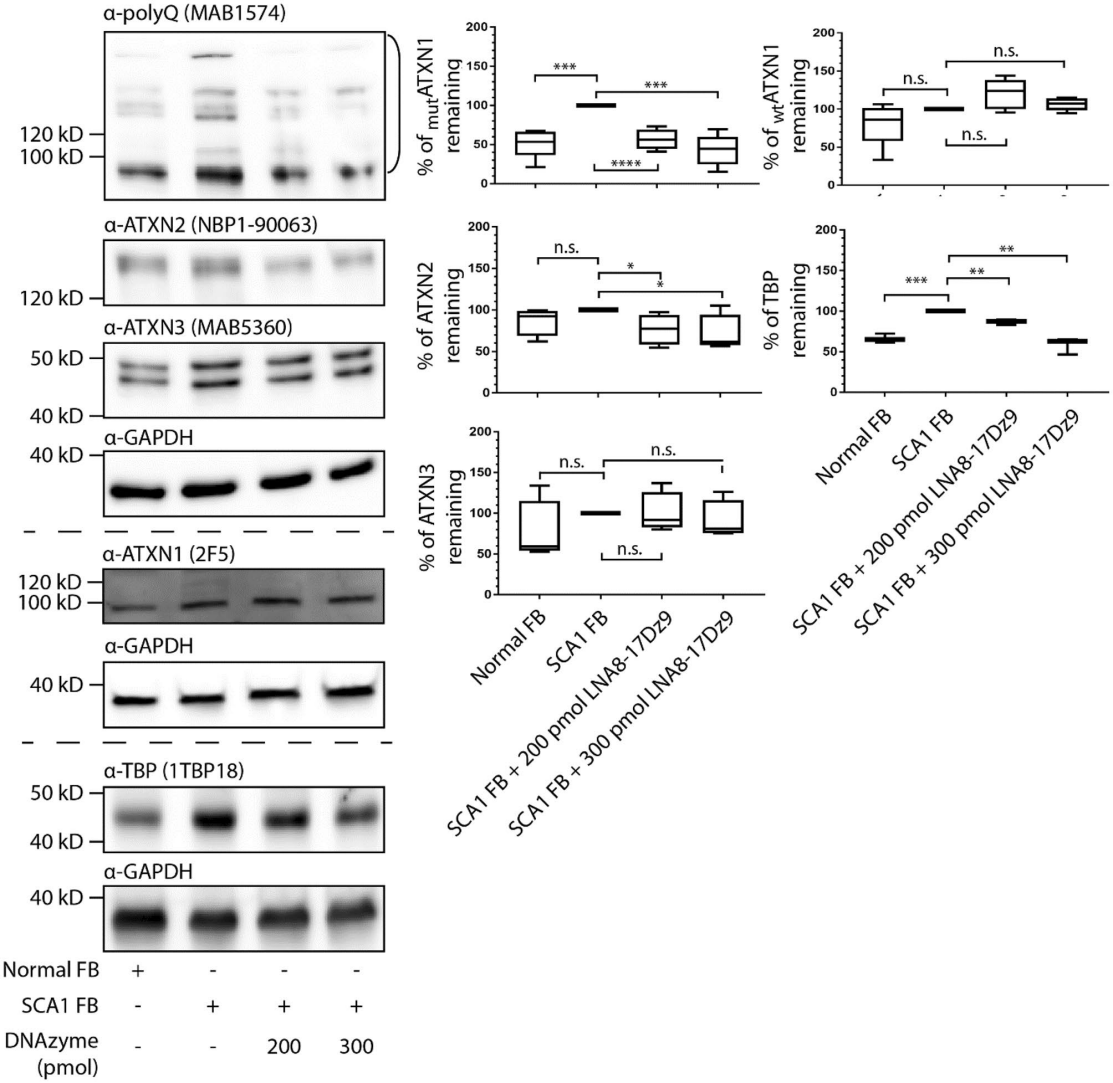
Allele specificity can be achieved by DNAzyme in SCA1 fibroblasts. Immunoblot and quantification of SCA1 fibroblasts nucleofected with 200 or 300 pmol LNA8-17Dz9 (*n* = 4). Protein bands used for quantification of mutant polyQ proteins are highlighted by the open bracket. FB – fibroblast

**Fig. 5:**
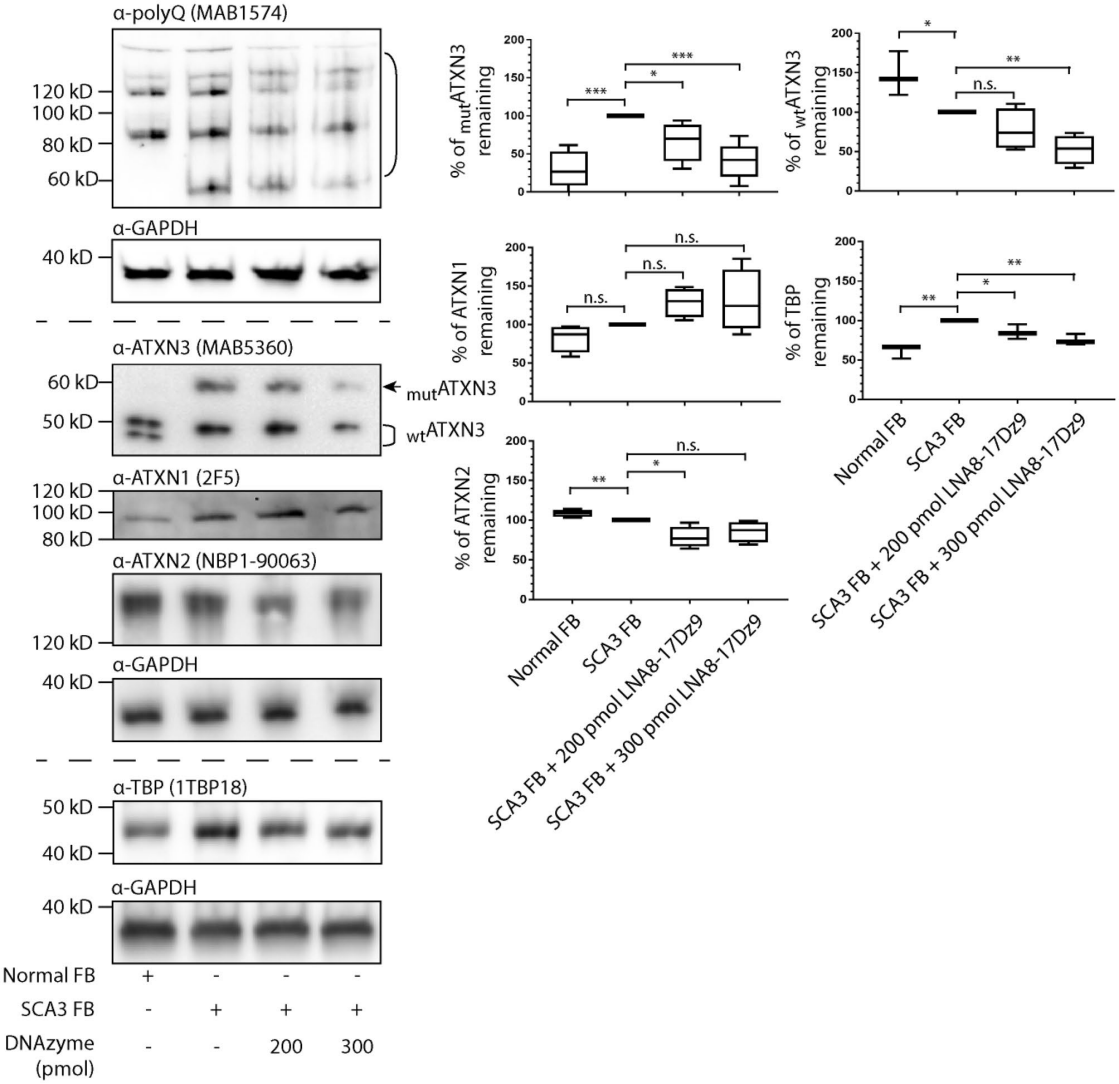
DNAzyme knocks down both wt and mutATXN3 in SCA3 fibroblasts. Immunoblot and quantification of SCA3 fibroblasts nucleofected with 200 or 300 pmol LNA8-17Dz9 (*n* = 4). Protein bands used for quantification of mutant polyQ proteins are highlighted by the open bracket. FB – fibroblast

To further investigate if LNA8-17Dz9 could achieve allele specificity, SCA1 and SCA3 patient-derived fibroblasts were nucleofected with either 200 or 300 pmol LNA8-17Dz9 for 48 h. In both cell lines, significant target knockdown *ATXN1* RNA in SCA1 and *ATXN3* RNA in SCA3 were observed (Fig. S12). In SCA1 fibroblasts, LNA8-17Dz9 preferentially reduces the mutATXN1 protein load (detected by the polyQ antibody as the ATXN1 antibody does not pick up mutATXN1) by 40–60% without affecting the wtATXN1 level (Fig. 4). It did not alter the wtATXN3 level but reduced the wtATXN2 level by approximately 30–40%. The LNA8-17Dz9 treatment reduced wtTBP to a level comparable to that in normal fibroblasts (Fig. 4). Even though ATXN3, ATXN2, and TBP all contain short CAG repeats, DNAzyme may affect their protein levels differentially (Fig. 4). Given that mutATXN1 may undergo aggregation, we assayed for insoluble fraction of protein post DNAzyme treatment and observed mutATXN1 knockdown by DNAzyme (Fig. S13). This observation is preliminary as there is not a good loading control in the insoluble fraction. In SCA3 fibroblasts, the LNA8-17Dz9 knocks down both wtATXN3 and mutATXN3 by 40–50%, closely tracking the results obtained in SCA3 iPSCs (Fig. 5 vs. Fig. 3E). Consistent with SCA1 cells, the wtATXN1 level was not affected by DNAzyme treatment while the wtATXN2 level was reduced by approximately 40% and the wtTBP to a similar level as observed in normal fibroblasts (Fig. 5). The reason as to why DNAzyme suppressed wtATXN3 in SCA3 fibroblasts but not in SCA1 fibroblasts could be due to differences in wt allelic copies, as SCA3 fibroblasts have only one wtATXN3 allele while the tested SCA1 fibroblasts have two. Such dosing effect could potentially be sensitive to DNAzyme treatment. In addition, the observed knockdown of both allele and non-allele selective manners requires further mechanistic investigations on the targeting moiety. To investigate the potential off-target effects of using repeat-based DNAzyme, we performed RNAseq analysis on mock- or LNA8-17Dz9-nucleofected wild-type fibroblast cells. By analyzing the expression of more than 60 published CAG-repeat containing mRNAs [80], we observed no significant transcriptomic off-target effects (Table S1).

**Fig. 6:**
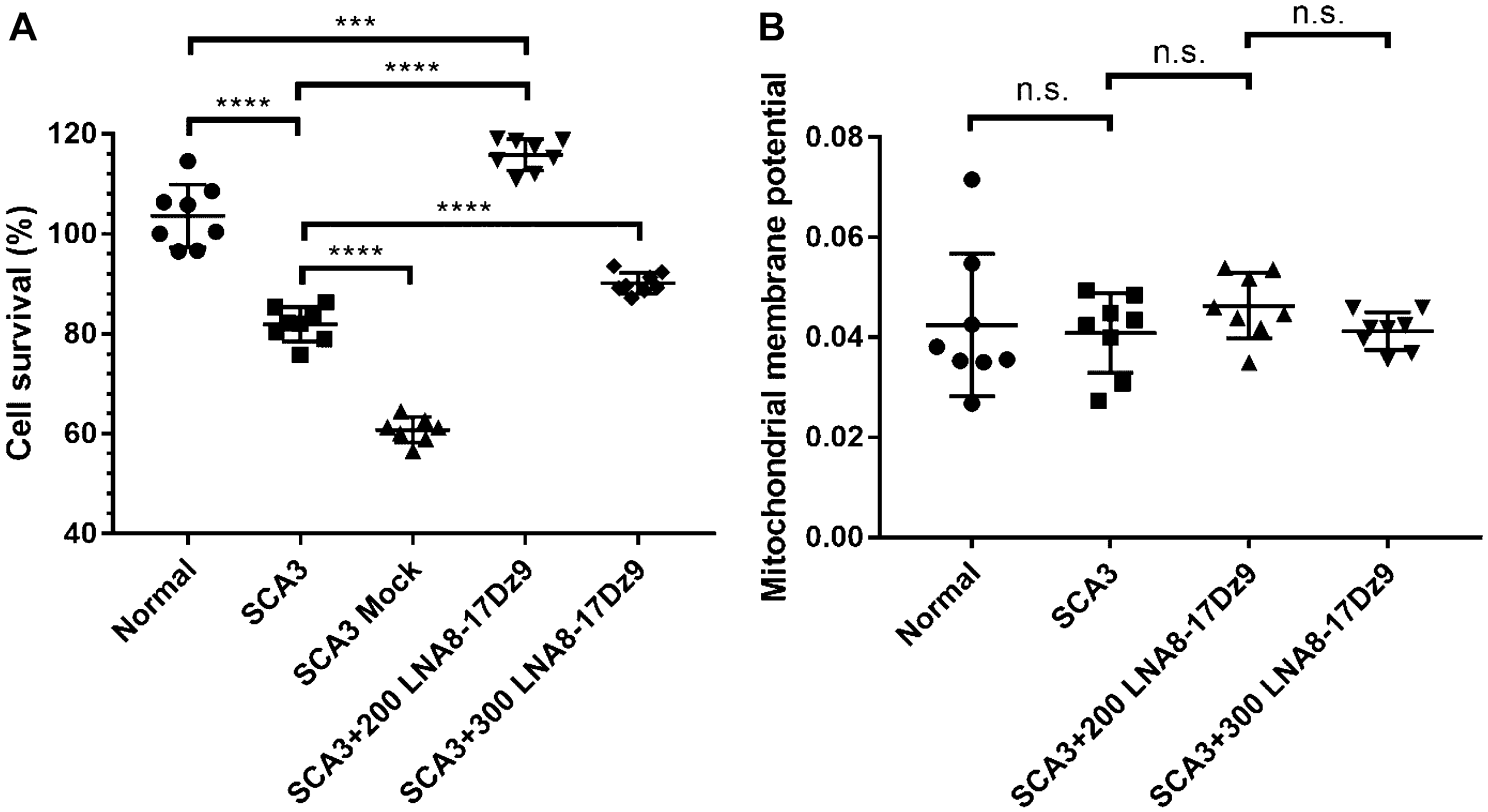
DNAzyme treatment rescues SCA3 fibroblast cell survival and had no detrimental effects on mitochondrial polarization. **A** Cell proliferation assay of nucleofected SCA3 fibroblasts (200 or 300 pmol LNA8-17Dz9) in comparison to untreated normal fibroblasts, untreated SCA3 cells, and mock-nucleofected SCA3cells (2 biological replicates each with 4 technical replicates). **B** Mitochondrial depolarization assay using JC-10 dye (2 biological replicates each with 4 technical replicates)

The DNAzyme-treated SCA3 fibroblasts were subject to further cell proliferation studies, given that potential off-target knockdown of wtATXN3, wtATXN2, and wtTBP was observed. To our surprise, the LNA8-17Dz9 treatment consistently improved SCA3 cell survival (even better survival than wt cells at low concentration, Fig. 6A). At a higher dosage of DNAzyme (300 pmol), SCA3 cell survival improved by > 30% compared to that of mock-nucleofected SCA3 cells (Fig. 6A). The above data show that the on-target reduction of mutant polyQ protein clearly benefits SCA3 cells. PINK1, PARKIN, and ATXN3 play important roles in mitochondrial maintenance and clearance [81, 82]. PINK1 is specifically activated by mitochondrial membrane potential depolarization, and recruits PARKIN to and activates PARKIN’s ubiquitinase activity on damaged mitochondria [83]. ATXN3 deubiquitinates PARKIN directly and reduces the extent of PARKIN ubiquitination in cells [81]. To see if knockdown of wtATXN3 in SCA3 fibroblasts by DNAzyme could off-set PARKIN’s ubiquitination on depolarized mitochondria and lead to accumulation of low-quality mitochondria, we measured mitochondrial membrane potential in DNAzyme-treated SCA3 fibroblasts and did not observe any change with respect to untreated or normal cells (Fig. 6B). As a result, we did not detect any oxidative DNA damage from mitochondrion-derived reactive oxygen species using 8-hydroxy-2-deoxyguanosine antibodies (8-OHdG, Fig. S14). Taken together, we demonstrate that allele-specific targeting of mutant polyQ protein can be achieved in SCA1 fibroblasts and the destruction of mutATXN3 outweighs the limited wtATXN3 reduction and significantly improves cell survival without compromising mitochondrial function in SCA3 fibroblasts.

We believe that enhanced delivery to target tissues/cells is an important area of research for translational application of DNAzyme. We explored two packaging/delivery methods in this study, namely, the liposome DCL64 formulation and DNA tetrahedron. Despite the significantly increased cellular uptake in both blood–brain barrier endothelial and neuroblastoma cells (Fig. S15), the DCL64-packaged DNAzyme only reduced the mutant polyQ protein load by 20% (Fig. S15F). Given that the fluorescently tagged DNAzyme is retained within cells (Fig. S15A–D), it is possible that the payload is not sufficiently released from the endosome, possibly due to a lack of ionizable cations in the formulation [84]. Various DNA tetrahedral nanoparticles have successfully delivered payload into glioma, kidney, or alveolar epithelial cells [85–87]. However, the biological effect after delivery remains largely uninvestigated. We show that DNA tetrahedron-packaged DNAzyme did not significantly knock down target protein in neuron-like cells, suggesting that unfacilitated endocytosis of DNA nanoparticle is insufficient to achieve cellular efficacy at least in our tested model (Fig. S15F). With recent advances in lipid nanoparticle formulation and cell penetrating peptide conjugation, it is possible to broaden DNAzyme’s biodistribution and bioavailability in animal models [88–91], even though a single or repeated injection of naked oligonucleotides already leads to a wide CNS spread in mice [30, 44].

### Stereotaxically Injected LNA8-17Dz9 Knocks Down High Molecular Weight ATXN3 In vivo

**Fig. 7:**
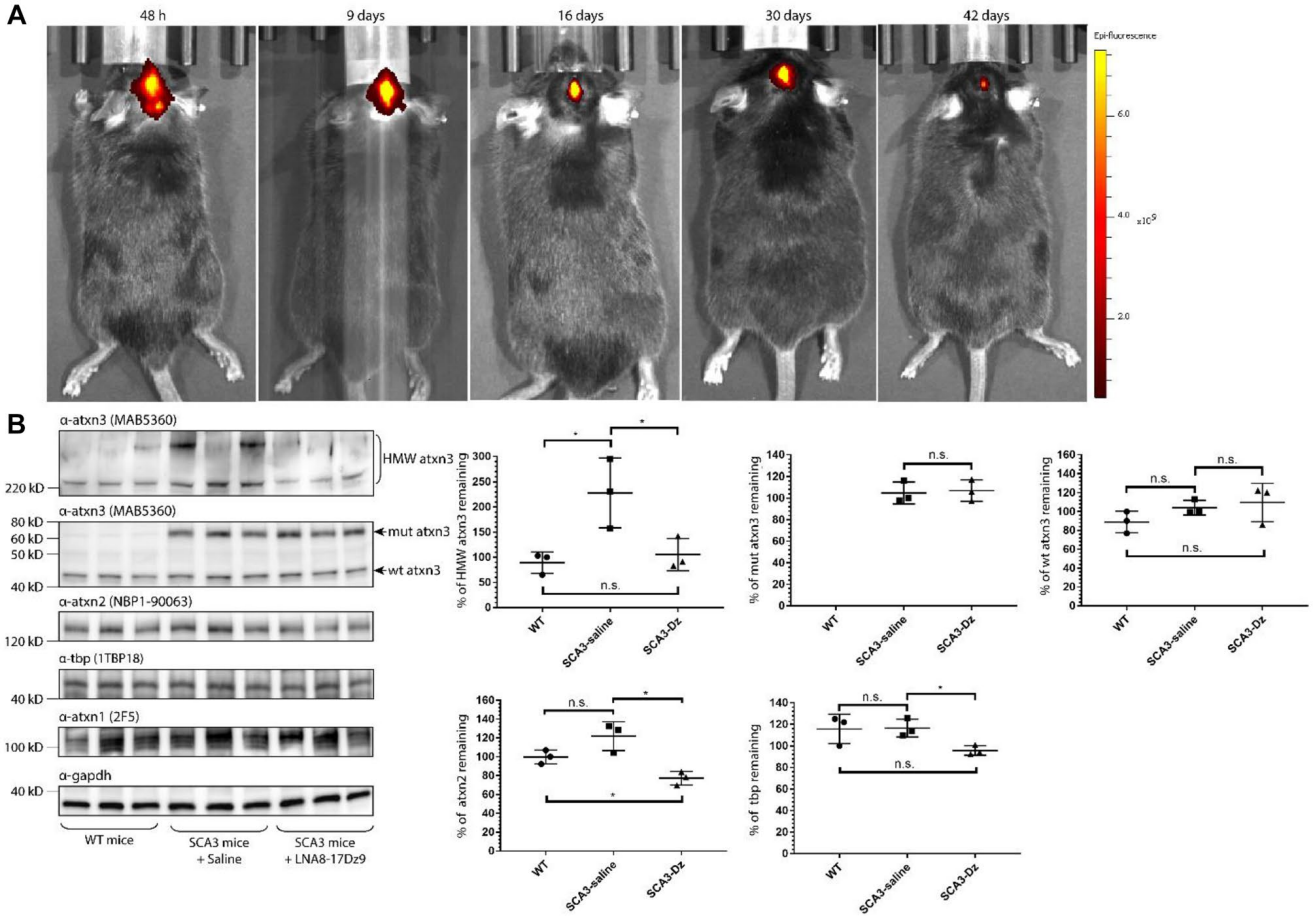
DNAzyme reduces HMW ATXN3 in MJD84.2 mouse brain. **A** I.V.I.S. imaging of low-dosage (25 μg) Cy5-labeled LNA8-17Dz9 stereotaxically injected in a SCA3 right lateral ventricle. All images were normalized to the same fluorescent scale. **B** Immunoblot and quantification of SCA3 mouse right cortex without treatment, with saline injection or with an intermediate dosage (400 μg) LNA8-17Dz9 (*n* = 3). The protein level of ATXN1 was not quantified due to promiscuity of the antibody

To evaluate the stability of LNA8-17Dz9 in vivo, we administered a low dosage (25 μg) of Cy5-labeled DNAzyme in the right ventricle of 6-week old SCA3 MJD84.2 mice by stereotaxic injection. Among several well established SCA3 mouse models, the transgenic MJD84.2 mouse was chosen for the following studies because the YAC construct harbors a human *ATXN3* gene (with Q3KQ80 repeats) under its native promoter and the mouse exhibits disease-relevant neuropathological phenotype and behavioral abnormalities from 4 weeks of age [92, 93]. I.V.I.S. imaging was performed 48 h to 42 days post injection and suggests that the low-dose LNA8-17Dz9 is detectable in mouse brain for at least 1 month (Fig. 7A). However, we cannot rule out the possibility that the total fluorescence signal may overestimate the quantity of intact Cy5-labeled LNA8-17Dz9, as partially cleaved DNAzyme and free Cy5 may be present. Six-week old SCA3 mice (*n* = 4) were subsequently injected with an intermediate dose (400 μg) of LNA8-17Dz9 in the same brain region. One month post injection, three of the four injected mice were harvested for immunoblotting and qRT-PCR and one for IF imaging (all from the right cortex). For comparison, age-matched WT mice and SCA3 mice injected with saline (*n* = 4 for each) were used as controls. All the DNAzyme-injected mice survived and showed no signs of toxicity (Table S2) or apparent gliosis (Fig. S16). By immunoblotting, we demonstrate that the LNA8-17Dz9 significantly reduced the HMW atxn3 accumulation [30, 34] by approximately 50% (Fig. 7B). At the tested dosage, we did not observe changes in the level of soluble, monomeric mut atxn3 and wt atxn3 but a 20% reduction of wt atxn2 (Fig. 7B). The wt tbp level post DNAzyme treatment was not significantly different from that of WT mouse (Fig. 7B), consistent with our observations in patient-derived fibroblast cells (Figs. 4 and 5). At an RNA level, we observed a general trend of reduction in the tested polyQ RNA (Fig. S17).

## Discussion

In this study, we have designed and optimized a repeat-based LNA-modified DNAzyme (LNA8-17Dz9) that can significantly reduce mutant polyQ RNA or protein load in several disease-related cell models and in one animal model. Our data provide the proof of principle that DNAzyme cleaves expanded CAG repeats of multiple polyQ diseases. The most significant difference between DNAzyme and other RNA silencing therapies is that it destroys target RNA without any auxiliary enzyme such as RNAse H or the RNA-induced silencing complex (RISC). High-resolution computational modeling of human RNase H1 bound to an RNA/DNA hybrid indicates that the scissile phosphate aligns with the cleavage pocket and is approximately 9–10 nt from either end of the target RNA [94]. This distance coincides with the mismatched region (the 15-nt catalytic core and the T·G mismatch) of the LNA8-17Dz9 (Fig. 1A), likely distorting the local interaction network within the cleavage pocket and rendering RNase H ineffective. This may be analogous to RNA interference where imperfectly matched double-stranded RNA disrupts Argonaute 2 active site interaction and subsequently hinders cleavage [95, 96]. In contrast to elevated cell death through RNase H recruitment by a repeat-based LNA gapmer [47], we observed improved cell proliferation after DNAzyme treatment (Fig. 6A). The ability of DNAzyme to cleave RNA without any protein component avoids overburdening cellular machinery.

Mutant protein knockdown by LNA8-17Dz9 is best achieved in neuronal cells (iNeuron and neuroblastoma, > 90%), followed by kidney embryonic cells (HEK293, 80–100%), patient-derived fibroblasts (SCA1 and SCA3, 40–60%), and patient-derived iPSCs (SCA3, 50%). We estimate the transfection efficiency of DNAzyme into each cell type is as follows: patient-derived fibroblasts (80–90%), kidney embryonic cells (80–90%), neuroblastoma cells (50–60%), patient-derived iPSCs (30–40%), and iNeurons (< 10%). A direct correlation between transfection efficiency and target knockdown in each cell type may be difficult, as in some cell types the target RNA/protein is endogenously expressed while in others ectopically expressed. In addition, the target RNA/protein expression level and/or accessibility to DNAzyme may vary significantly in different cell types. And it is difficult to assess how efficiently DNAzymes escape from the endosome in different cell types. However, the greatest knockdown in neuronal cells infers that LNA8-17Dz9 is suitable for therapeutic application to neurodegenerative disorders. Previous ASO studies suggest that complete inhibition of mutant protein is not necessary and partial non-allelic reduction of mutHTT by 20–40%, mutATXN1 by 20% and mutATXN3 by 30% in mouse brain could already reverse molecular, phenotypic, and/or behavioral defects [30, 44, 97, 98]. We further demonstrate in a SCA3 mouse model that stereotaxically administered LNA8-17Dz9 (1) is stable for at least 1 month in mouse brain (tested at a low dosage), (2) neither shows toxic effects nor causes early death (tested at an intermediate dosage), and (3) reduces the HMW ATXN3 proteins by approximately 50% (tested at an intermediate dosage). We hypothesize that by increasing the administered dose of DNAzyme (> 750 μg per animal) more efficient target RNA/protein knockdown can be achieved by promoting the bioavailability in local tissue. Further examination of off-target effects, by performing RNAseq using mouse brains, molecular and behavioral rescue, and toxicity, by performing acute and long-term toxicological profiles, will be carried out using a larger sample size.

One of the main drawbacks of targeting CAG repeat RNA is the potential lack of specificity—short CAG repeats are present in the normal target allele as well as > 1000 genes [99]. By performing RNAseq on mock- and LNA8-17Dz9-nucleofected normal fibroblasts, we observed no significant alteration of > 60 published mRNA containing different CAG repeat lengths at a transcriptomic level (Table S1). The rationale for allele-specific knockdown using LNA8-17Dz9 is that the expanded CAG RNA contains more binding sites and that its 3D folding is energetically different from that of short CAG repeats for targeting [48]. Indeed, we successfully demonstrate that LNA8-17Dz9 specifically knocks down 40–60% mutATXN1 in SCA1 fibroblasts without affecting the endogenous ATXN1 or ATXN3 protein levels (Fig. 4A). Allele-specific targeting of mutATXN1 by LNA8-17Dz9 is beneficial because mice lacking Ataxin1 display learning deficits [100]. However, we did observe a 20–40% knockdown of endogenous ATXN2 and TBP, which is consistently reproduced in both SCA1 and SCA3 fibroblasts (Figs. 4 and 5). The 20–40% ATXN2 knockdown should be well-tolerated because *Atxn2*-KO mice display normal weight, lifespan, exploratory patterns, and spatial learning, and *Atxn2*-knockdown TDP43 transgene mice exhibit increase lifespan by 35% [101, 102]. The 20–40% knockdown of TBP in SCA1/SCA3 fibroblasts brings the protein level similar to normal fibroblasts (Figs. 4 and 5), which is consistently reproduced in the SCA3 mouse model (Fig. 7B). In SCA3 fibroblasts and iPSCs, ATXN3 was knocked down by 40–60% non-allele-specifically by LNA8-17Dz9 (Figs. 3E and 4B). Given that Ataxin3 is not essential for mouse viability and fertility [75, 76], the observed reduction of ATXN3 should be well-tolerated. However, we do not anticipate detrimental consequences because (1) DNAzyme treatment improves SCA3 cell survival without affecting mitochondrial function or p62-dependent autophagy (Figs. 5 and S11), (2) DNAzyme-treated fibroblasts show no significant RNAseq difference of > 60 CAG-containing mRNA when compared to mock-treated cells (Table S1), (3) knockdown of endogenous genes expressing short CAG repeats by an LNA ASO in WT mice did not generate toxicity or attenuate cell function [103], and (4) the DNAzyme-treated SCA3 mice did not show acute toxicity or death.

LNA8-17Dz9 is restricted to cleavage between adenine (A) and guanine (G). The 10–23 DNAzyme (10-23Dz) cleaves any purine–pyrimidine (RY, R = A or G; Y = U or C) junction and may be tried for a wide variety of diseases [104]. The RY cleavage efficiency is in the order of AU = GU ≥  ≥ GC >  > AC [105]. The 10-23Dz may cleave the expanded CUG repeat in myotonic dystrophy 1 (DM1 [106]) and Fuchs endothelial corneal dystrophy (FECD [107]) at GC sites, the intronic CCUG repeat in myotonic dystrophy 2 (DM2 [108]) and GGGGCC repeat in C9orf72-amyotrophic lateral sclerosis/ frontotemporal dementia (ALS/FTD [109]) at GC sites, the CGG repeat in Fragile X–associated tremor ataxia syndrome (FXTAS [110]) and oculopharyngodistal myopathy (OPDM [111]) at GC sites, and the AU-rich repeats in SCA10 [112], SCA31 [113], and SCA37 [114] at AU sites. Combining 8-17DZ and 10-23Dz, DNAzymes can target most, if not all, expanded microsatellite repeats in gain-of-function disorders.

In conclusion, using a single therapeutic DNAzyme to treat potentially a panel of polyQ neurodegenerative diseases is attractive, as it greatly simplifies the processes of rational design, pharmacodynamic and kinetic validation, and manufacture. More focused chemical modifications can be tailored to the same agent without variations arising from sequence changes. Moreover, repeat-based targeting may offer unexpected advantages over strategies that target unique sequences (or single-nucleotide polymorphisms, SNPs). This is because the potency of targeting unique sequences is greatly influenced by RNA processing dynamics and isoform structure, and the potency is consistently reduced in SCA3 and SBMA mice derived from cDNA transgenes but not so in animals derived from full-length transgenes [34, 115, 116]. Although DNAzyme can be reprogrammed to recognize SNPs, extensive genotyping needs to be performed to identify SNPs sufficiently present in the majority of patients. For instance, it has been estimated that 5–7 allele-specific oligonucleotides are needed to cover 75–85.6% of HD patients [117–119]. Combining multiple DNAzymes into a single therapy with regulatory approval may be difficult. It could be challenging when no SNPs associated with polyQ mutation can be identified (such as SCA2) [120], when the SNP-targeting oligonucleotide does not show intended allelic selectivity [121], or when patients are homozygous for the targeted SNPs. Hence, repeat-based DNAzyme provides new hope for currently incurable polyQ neurodegeneration and its reprogrammability can extend to other diseases with RNA pathology or etiology.

## Supplementary Information

Below is the link to the electronic supplementary material.

Supplementary file1 (DOCX 5275 KB)

Supplementary file2 (PDF 432 KB)

Supplementary file3 (PDF 432 KB)

Supplementary file4 (PDF 433 KB)

Supplementary file5 (PDF 432 KB)

Supplementary file6 (PDF 433 KB)

Supplementary file7 (PDF 433 KB)

Supplementary file8 (PDF 431 KB)

Supplementary file9 (PDF 433 KB)

Supplementary file10 (PDF 436 KB)

Supplementary file11 (PDF 436 KB)

Supplementary file12 (PDF 435 KB)

## Data Availability

The datasets used and/or analyzed in the current study are available from the corresponding author on reasonable request.

## Abbreviations

8-OHdG: 8-Hydroxy-2-deoxyguanosine
ASO: Antisense oligonucleotide
ATXN1: Ataxin 1
ATXN2: Ataxin 2
ATXN3: Ataxin 3
ATXN7: Ataxin 7
CNS: Central nervous system
DPPC: 1,2-Dipalmitoyl-sn-glycero-3-phosphocoline
DRPLA: Dentatorubropallidoluysian atrophy
Dz: DNAzyme
eGFP: Enhanced green fluorescent protein
HD: Huntington’s disease
HMW: High molecular weight
hNGN2: Human neurogenin 2
HTT: Huntingtin
HTTex1: HTT exon 1
i.c.v.: Intracerebroventricular
IF: Immunofluorescence
iNeuron: Induced neuron
iPSC: Induced pluripotent stem cell
I.V.I.S: In vivo imaging system
LNA: Locked nucleic acid
Mut: Mutant
PNA: Peptide nucleic acid
polyQ: Polyglutamine
P/S: Penicillin–streptomycin
RAN: Noncanonical repeat-associated non-ATG
SBMA: Spinobulbar muscular atrophy
SCA: Spinocerebellar ataxia
TBP: TATA binding protein
Wt: Wildtype
