# Supplementary material for: DNAzyme Cleavage of CAG Repeat RNA in Polyglutamine Diseases
Source: Neurotherapeutics. 2021 Jun 23;18(3):1710–28. doi: 10.1007/s13311-021-01075-w (PMC8609077; doi:10.1007/s13311-021-01075-w)
Supplement: Supplementary file 1 — Supplementary file1 (DOCX 5275 KB) [file 13311_2021_1075_MOESM1_ESM.docx]

**Supplementary Figures**


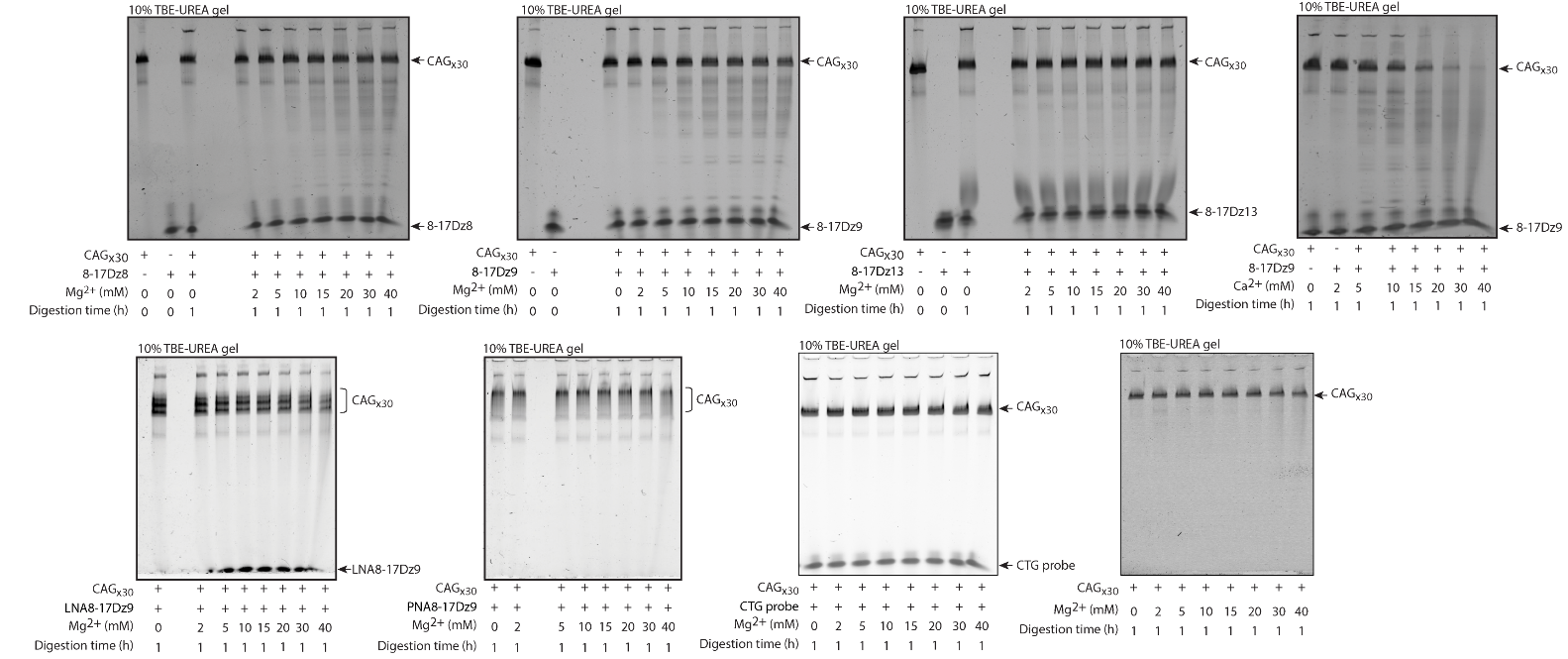


**Fig. S1. Representative gels used for biochemical cleavage measurement in Fig. 1B.**


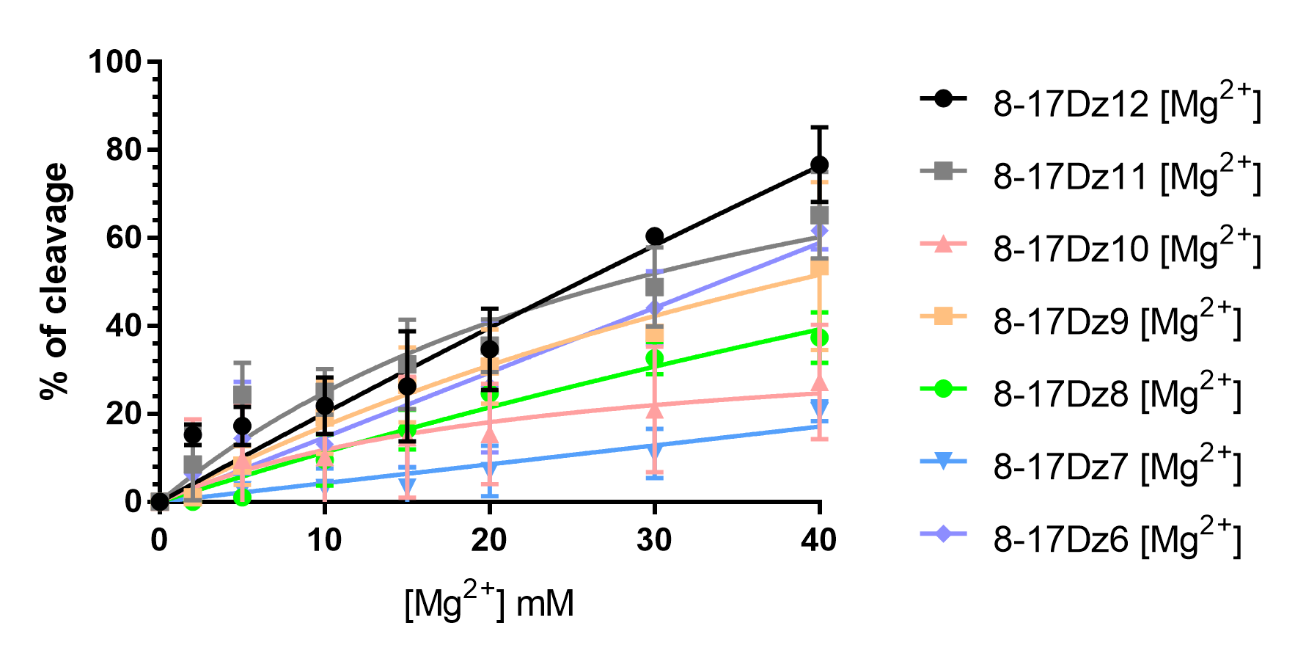


**Fig. S2.** **Alternations of RNA binding arm’s length change the catalytic property of 8-17Dz.** Reactions were performed under different Mg^2+^ concentrations (mM) at 37 °C for 1 h using *in vitro* transcribed CAG_x30_ RNA (n = 3).


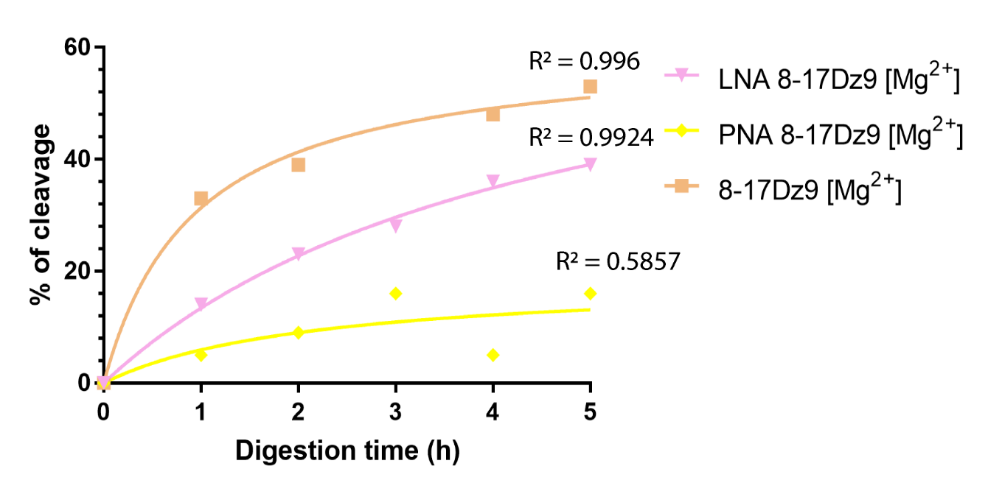


**Fig. S3. 8-17Dz cleaves RNA target under low ionic conditions.** Reactions were performed at 37 °C for 5 h using *in vitro* transcribed CAG_x30_ RNA and 5 mM Mg^2+^.


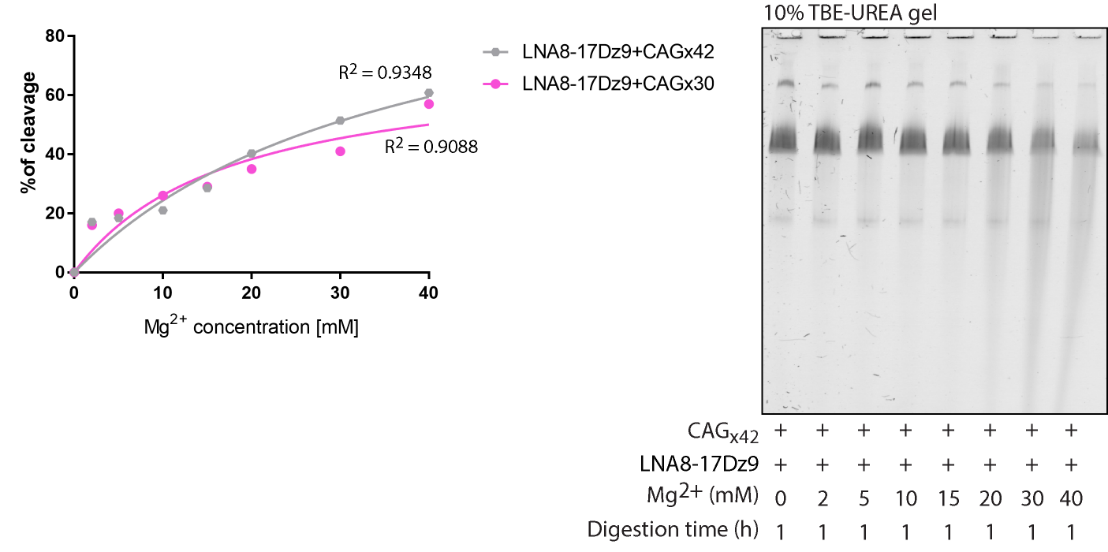


**Fig. S4. LNA8-17Dz9 cleaves both CAGx30 and CAGx42 repeats with comparable efficiency biochemically.** Reactions were performed at 37 °C for 1 h using *in vitro* transcribed CAG_x42_ RNA and different concentrations of Mg^2+^. The CAG_x42_ repeats were cloned into the pcDNA3.1 backbone for in vitro transcription. Generation of longer repeats is limited by the synthesis power commercially.


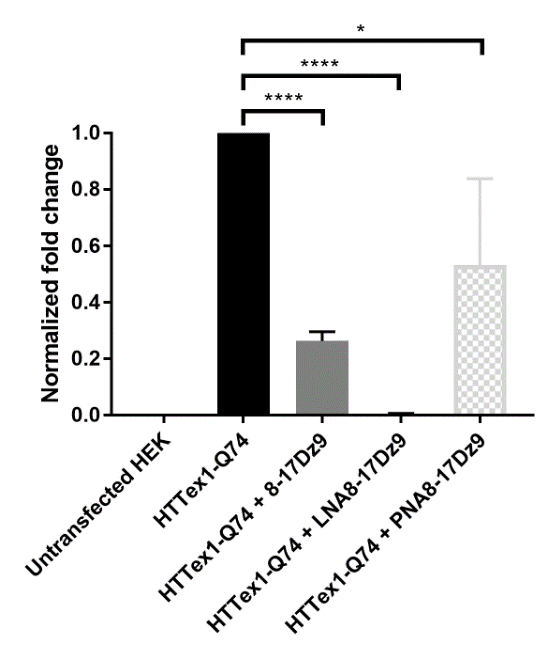


**Fig. S5. *HTTex1-Q74* mRNA expression after treatment with three 8-17Dz9 designs.** Total RNA was extracted 48 h post transfection in HEK293 cells, reverse transcribed and used in qRT-PCR. RNA expression was normalized against *GAPDH* mRNA and expressed as fold change using Bio-Rad CFX Manager 3.1. Three biological replicates each with three technical replicates were used for calculation.


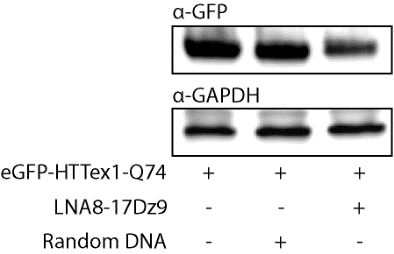


**Fig. S6. HTTex1-Q74 protein knockdown is not due to transfection competition from LNA8-17Dz9.** Immunoblot of HEK293 cells co-transfected with GFP-HTTex1-Q74 and LNA8-17Dz9 or a random DNA fragment (of the same length) after 48 h.


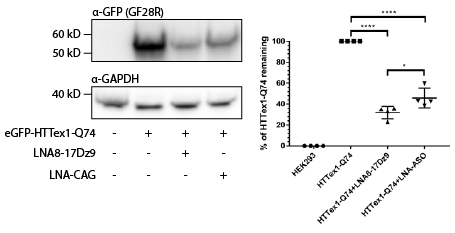


**Fig. S7. LNA8-17Dz9 is more efficient at clearing the expanded eGFP-HTTex1-Q74 than a repeat-based LNA-ASO.** The ASO has the same binding arms and LNA modifications as the LNA8-17Dz9 but minus the catalytic loop. HEK293 cells were co-transfected with eGFP-HTTex1-Q74 and 200 pmol of LNA8-17Dz9 or ASO for 48 h before immunoblotting analysis.


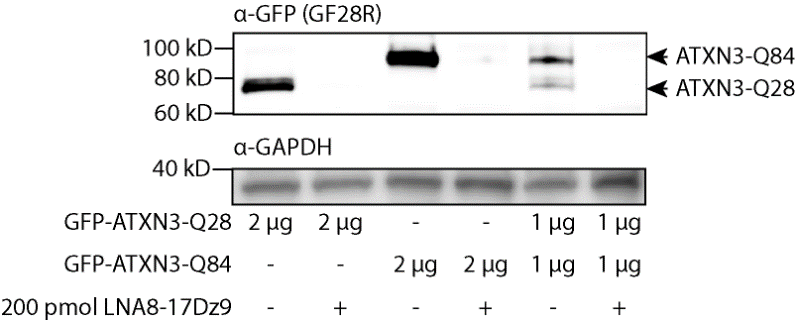


**Fig. S8.** **LNA 8-17Dz9 cleaves both GFP-tagged ATXN3-Q28 and ATXN3-Q84 in HEK293 cells.** HEK293 cells were transfected with 2 µg of ATXN3-Q28, 2 µg of ATXN3-Q84, or 1 µg of ATXN3-Q28 plus 1 µg of ATXN3-Q84 with or without 200 pmol LNA 8-17Dz9. Immunoblot was performed 48 h post transfection.


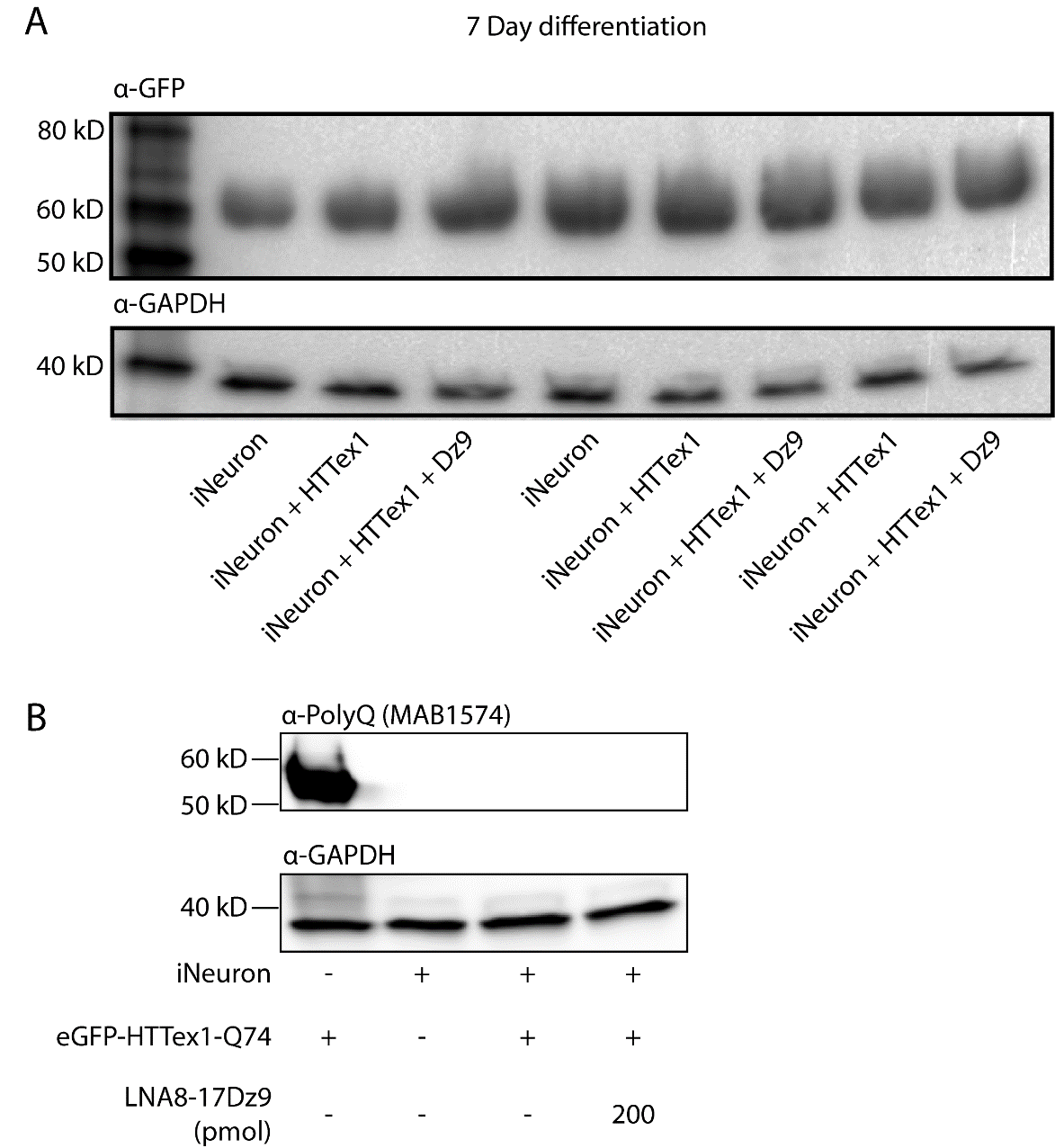


**Fig. S9. HTTex1-Q74 expression is completely lost on differentiation day 7 in iNeurons (n = 3).**

**
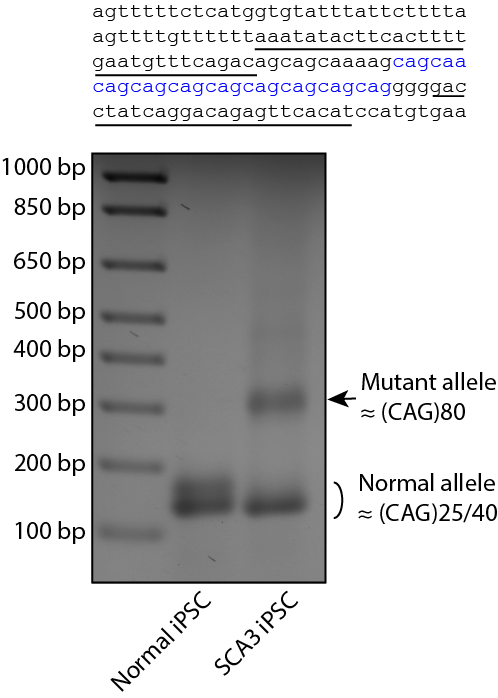
**

**Fig. S10. Determination of CAG repeat size in a patient-derived SCA3 iPSC.** PCR amplification of the *ATXN3* repeat region (highlighted in blue) was performed with primers (underlined sequence) and LongAmp (NEB) following the following thermo cycle: 95 °C 1 min, (95 °C 30 sec, 58 °C 30 sec, 65 °C 1 min)x 35, 65 °C 10 min, 4 °C hold.


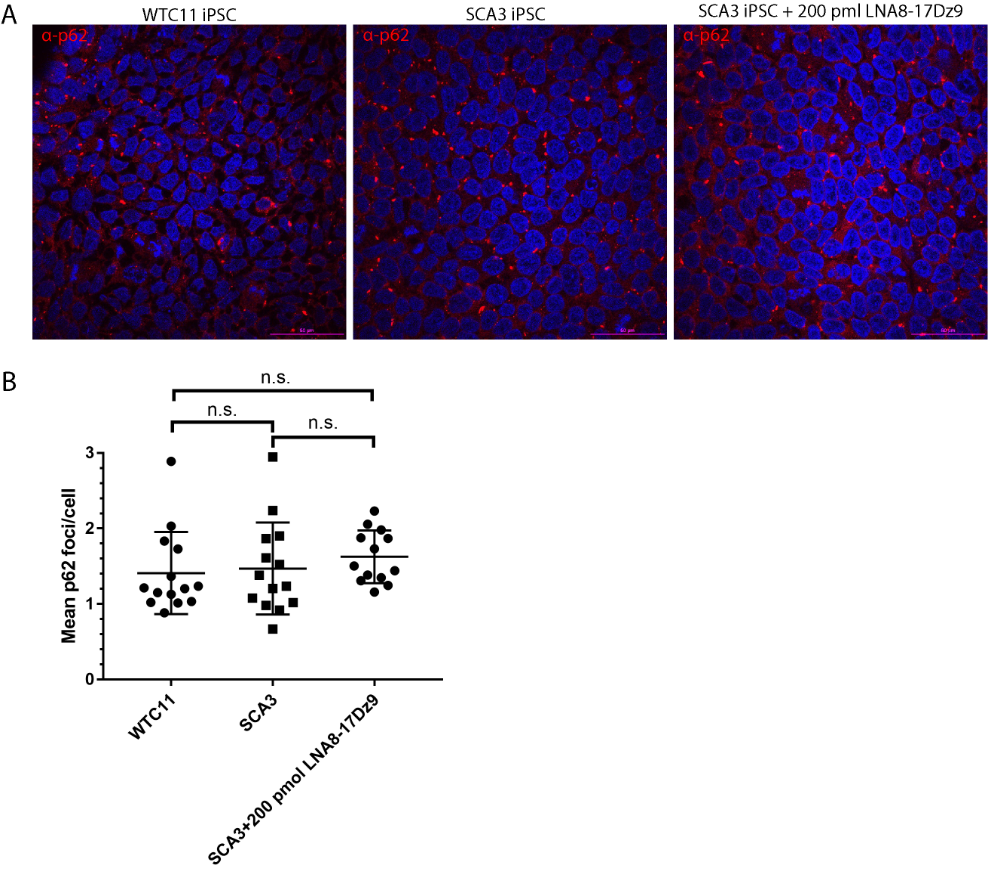


**Fig. S11. p62-dependent aggresome level is unaltered with DNAzyme treatment.** (A) IF against p62 in normal WTC11, untreated SCA3 and DNAzyme-treated SCA3 iPSCs (3 biological samples, 3266 cells were used for quantifying p62 aggresome in WTC11 iPSC, 3473 cells in untransfected SCA3 iPSC, and 2355 cells in transfected SCA3 iPSC). (B) Quantification of (A).


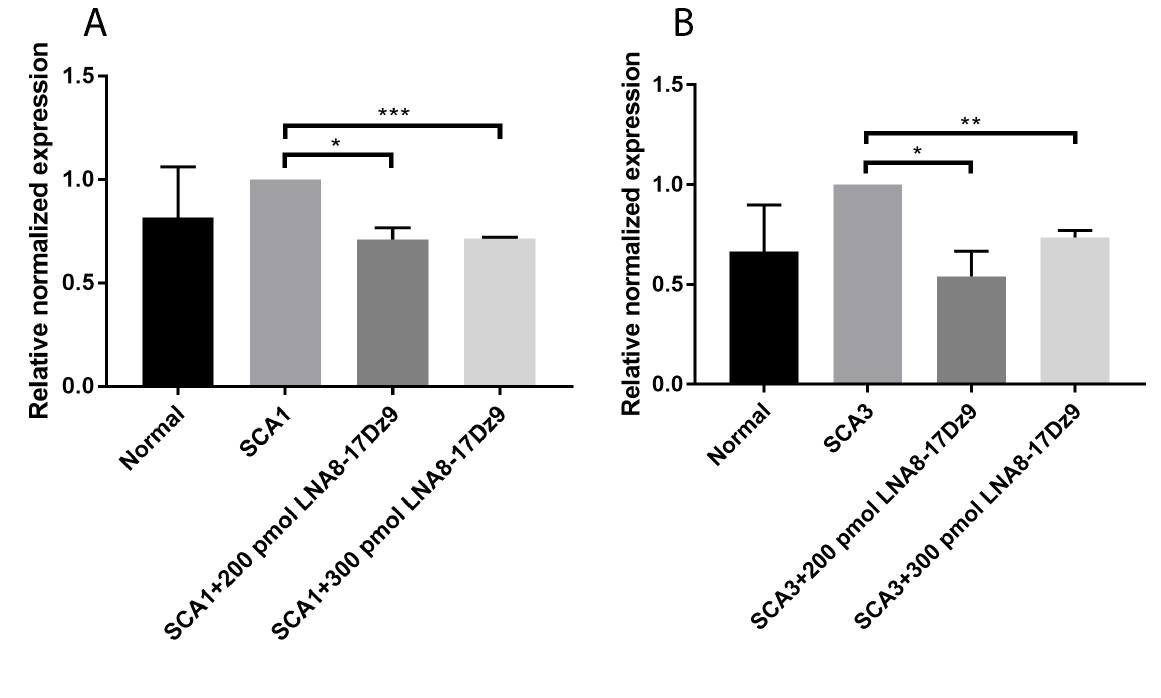


**Fig. S12. qRT-PCR of target RNA knockdown by DNAzyme in SCA1 and SCA3 fibroblasts.** (A) Normalized *ATXN1* RNA fold change in normal, untreated SCA1 and DNAzyme-treated SCA1 fibroblasts (2 biological samples each with 3 technical samples). (B) Normalized *ATXN3* RNA fold change in normal, untreated SCA3 and DNAzyme-treated SCA3 fibroblasts (2 biological samples each with 3 technical samples). The primers used for the qRT-PCR reactions were adopted from [1, 2].


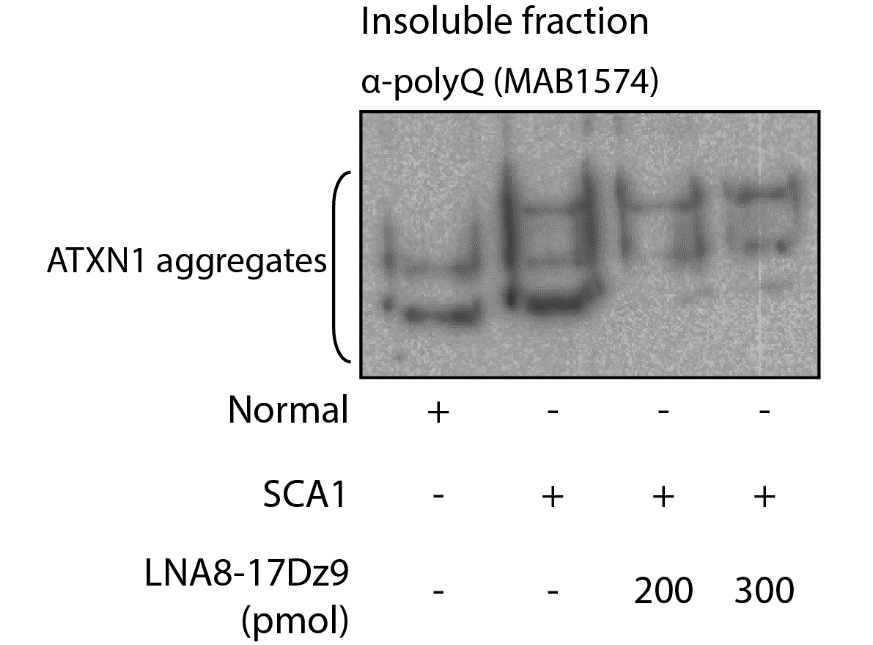


**Fig. S13. DNAzyme reduces mutATXN1 protein in the insoluble fraction of SCA1 fibroblast cells.**

**
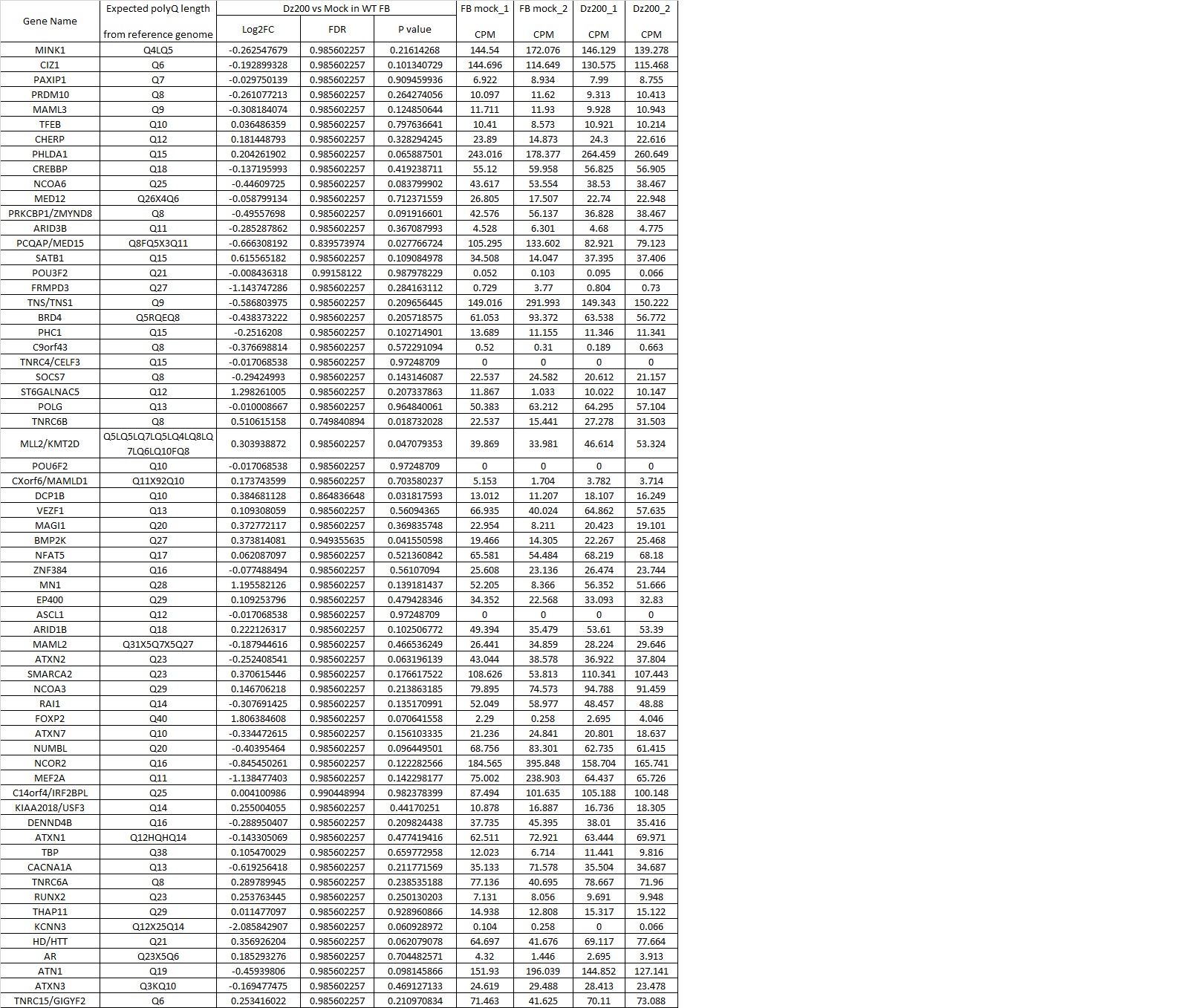
**

**Table. S1. Compared to the mock-treated normal fibroblast cells (FB mock), LNA8-17Dz9 treated normal cells (Dz200) do not show significant changes in expression of mRNA containing different CAG repeat lengths.** Log2FC, FDR and p-value were calculated, and significance level was determined at FDR < 0.25 and p-value < 0.05, or | Log2FC | > 1.5 and FDR < 0.05.


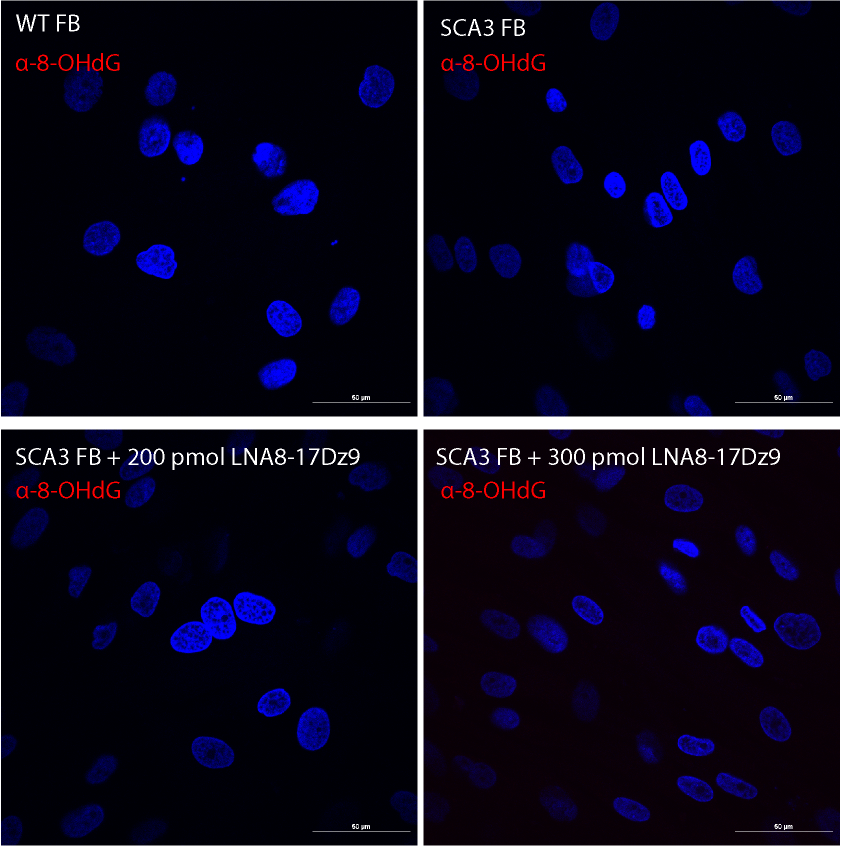


**Fig. S14. DNAzyme treatment does not change reactive oxygen species (ROS) induced DNA damage.** IF was performed against 8-OHdG (n = 3).


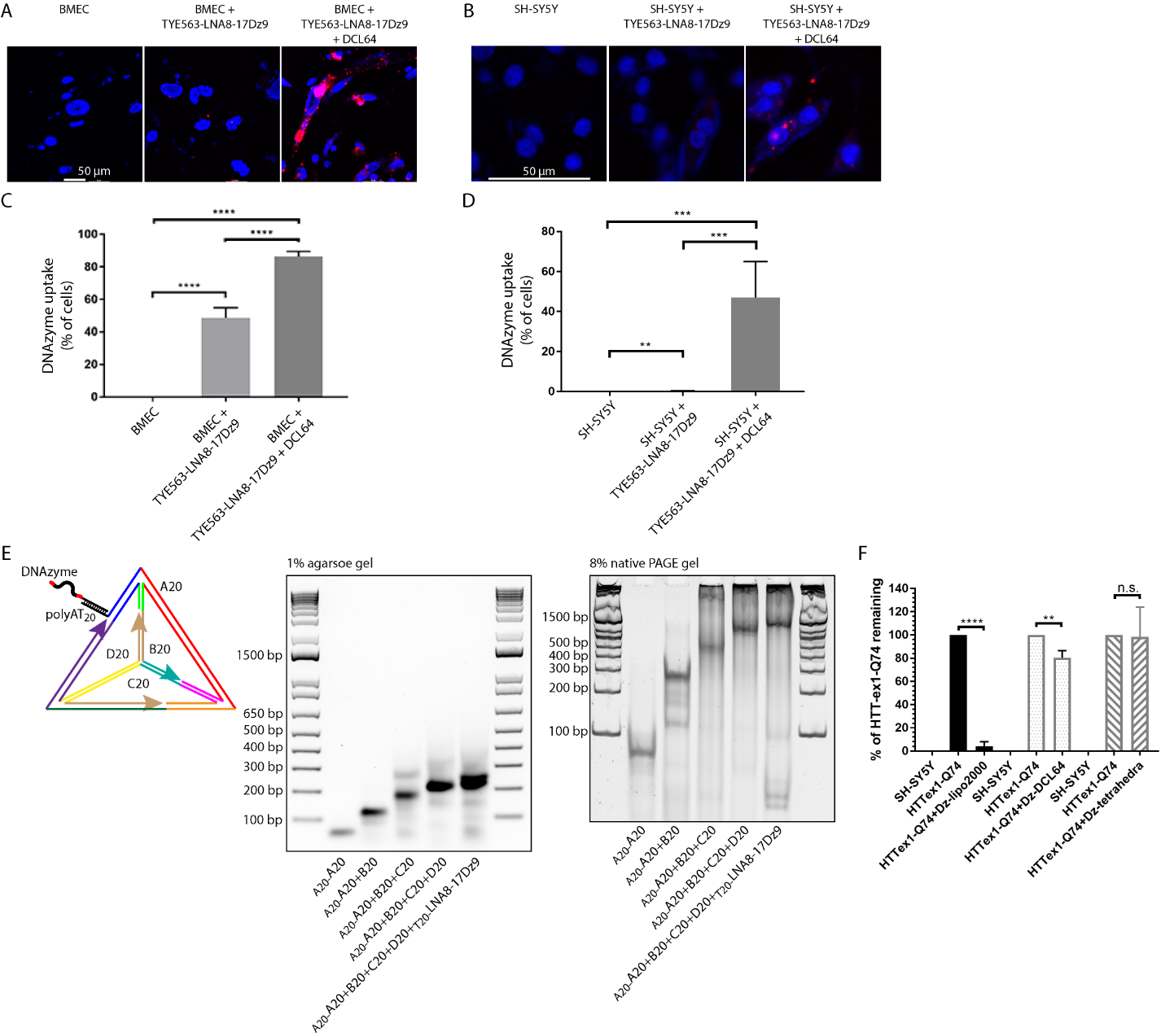


**Fig. S15. Packaging and delivery of LNA8-17Dz9 with liposome DCL64 or DNA tetrahedron.** Retention of TYE563-LNA8-17Dz9 in BMECs with or without DCL64 packaging (A and C, 2 biological replicates for each condition, 52 cells analyzed for BMEC+DNAzyme, 76 cells analyzed for BMEC+DNAzyme+DCL64). Retention of TYE563-LNA8-17Dz9 in SH-SY5Y cells with or without DCL64 packaging (B and D, 2 biological replicates for each condition, >300 cells analyzed for SH-SY5Y+DNAzyme or SH-SY5Y+DNAzyme+DCL64). (E) DNA tetrahedra with 20-nucleotide edge lengths (A-D20) were assembled and analyzed on agarose and native PAGE gels. The A20 strand has a polyA_20_ overhang for hybridization with a polyT_20_ tail on LNA8-17Dz9. (F) HTTex1-Q74 protein reduction by LNA8-17Dz9 delivered via lipofectamine 2000, liposome DCL64 or DNA tetrahedra (3 biological replicates for each condition).


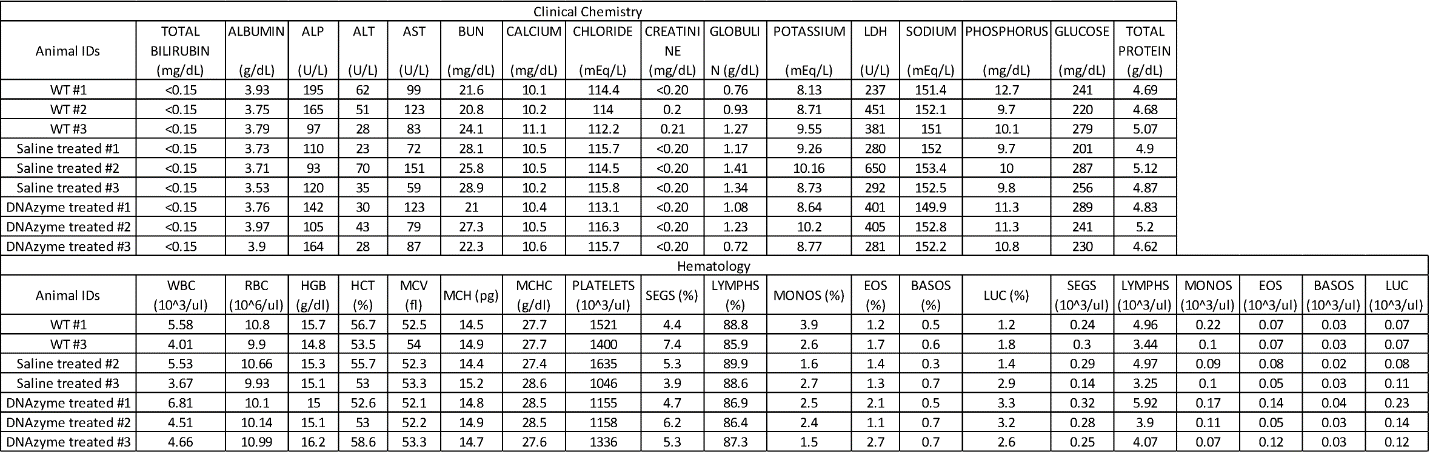


**Table. S2. Toxicology report of DNAzyme-treated SCA3 mice in comparison to WT and saline-treated SCA3 mice.**

**
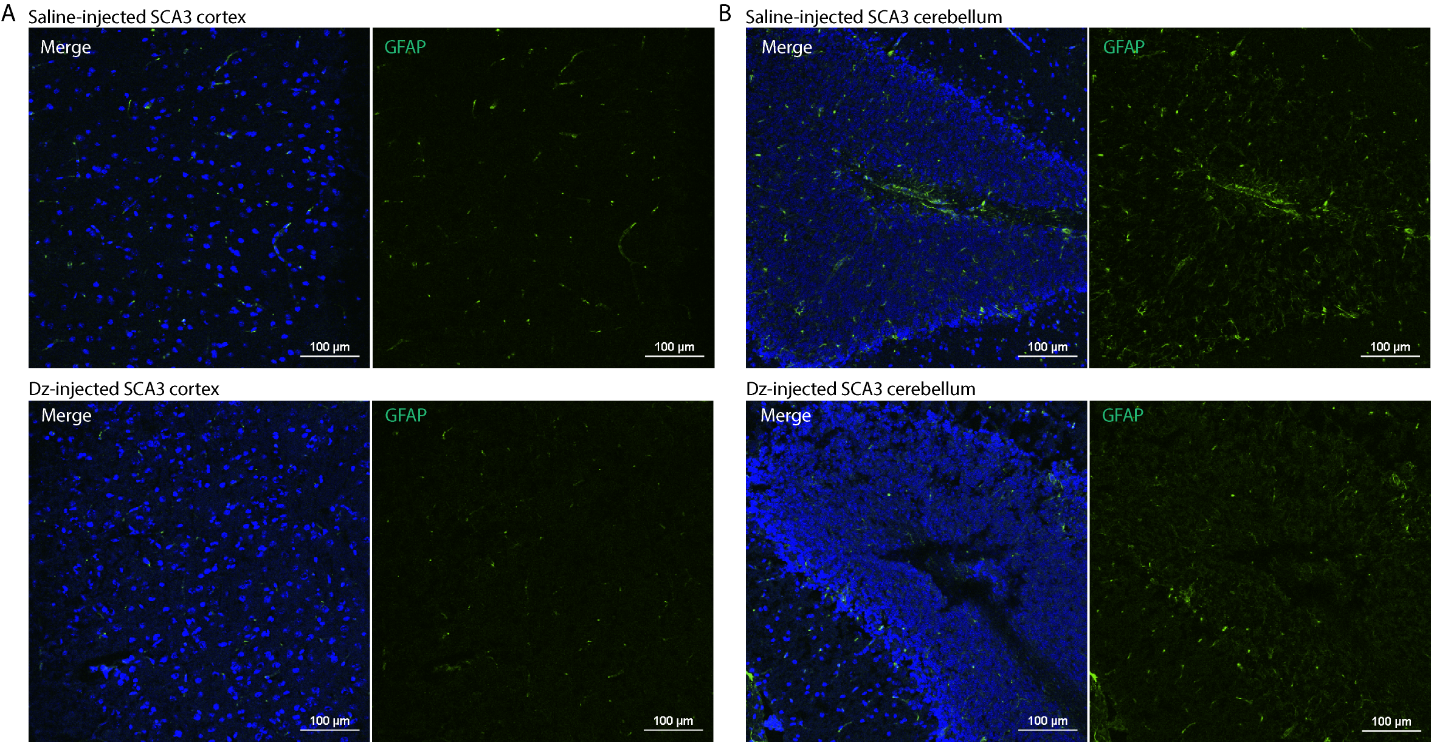
**

**Fig. S16. IF imaging of the gliosis marker GFAP in saline-treated or DNAzyme-treated SCA3 mouse brain.** (A) IF of cortex and (B) IF of cerebellum (1 brain with 3 sections for each right sagittal brain section examined).


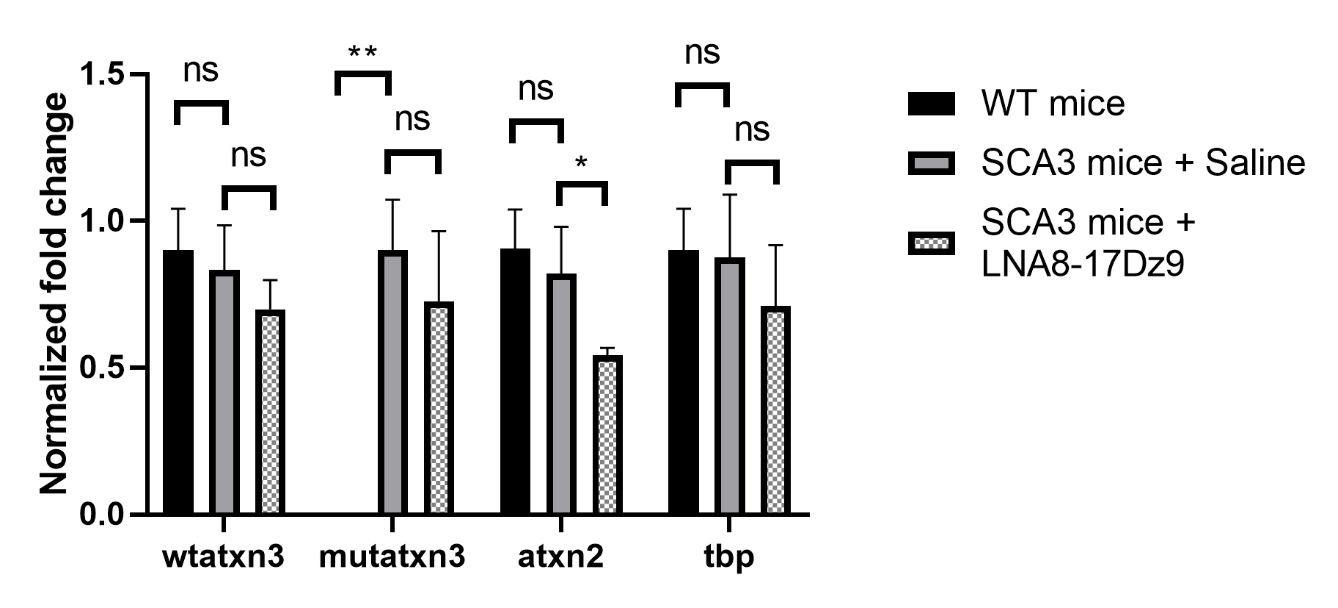


**Fig. S17. qRT-PCR of total RNA extracted from right mouse brain cortex used in Fig. 7B.** mutATXN3 was detected using a transgene specific primer pair.


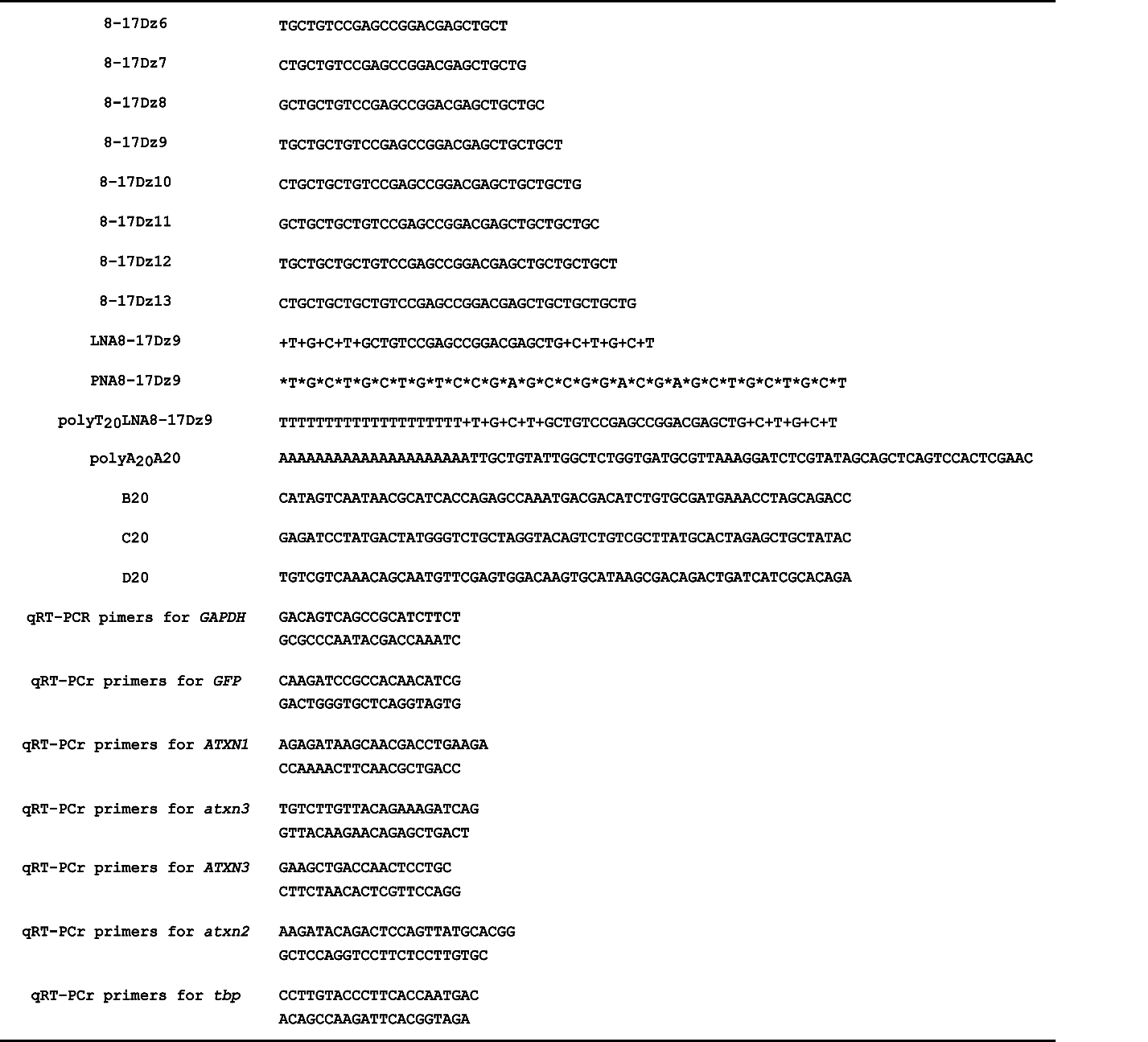


**Table S3. List of oligonucleotides used in this study.** The primers used for the qTR-PCR reactions were adopted from [1-4].

**References:**

1. Moore LR, Rajpal G, Dillingham IT, Qutob M, Blumenstein KG, Gattis D, et al. Evaluation of Antisense Oligonucleotides Targeting ATXN3 in SCA3 Mouse Models. Mol Ther Nucleic Acids. 2017; 7: 200-10.

2. Rousseaux MWC, Tschumperlin T, Lu HC, Lackey EP, Bondar VV, Wan YW, et al. ATXN1-CIC Complex Is the Primary Driver of Cerebellar Pathology in Spinocerebellar Ataxia Type 1 through a Gain-of-Function Mechanism. Neuron. 2018; 97: 1235-43 e5.

3. Gong H, Sun L, Chen B, Han Y, Pang J, Wu W, et al. Evaluation of candidate reference genes for RT-qPCR studies in three metabolism related tissues of mice after caloric restriction. Sci Rep. 2016; 6: 38513.

4. Li PP, Sun X, Xia G, Arbez N, Paul S, Zhu S, et al. ATXN2-AS, a gene antisense to ATXN2, is associated with spinocerebellar ataxia type 2 and amyotrophic lateral sclerosis. Ann Neurol. 2016; 80: 600-15.
